# Preparation of Solid Lipid Nanoparticles and Nanostructured Lipid Carriers for Drug Delivery and the Effects of Preparation Parameters of Solvent Injection Method

**DOI:** 10.3390/molecules25204781

**Published:** 2020-10-18

**Authors:** Van-An Duong, Thi-Thao-Linh Nguyen, Han-Joo Maeng

**Affiliations:** 1 Ho Chi Minh City University of Technology (HUTECH), Ho Chi Minh City 700000, Vietnam; dv.an@hutech.edu.vn; 2 College of Pharmacy, Gachon University, 191 Hambakmoe-ro, Yeonsu-gu, Incheon 21936, Korea

**Keywords:** lipid nanoparticles, solvent injection, entrapment efficiency, diffusion, aqueous phase, organic phase, solid lipid, liquid lipid, emulsifier

## Abstract

Solid lipid nanoparticles (SLNs) and nanostructured lipid carriers (NLCs) have emerged as potential drug delivery systems for various applications that are produced from physiological, biodegradable, and biocompatible lipids. The methods used to produce SLNs and NLCs have been well investigated and reviewed, but solvent injection method provides an alternative means of preparing these drug carriers. The advantages of solvent injection method include a fast production process, easiness of handling, and applicability in many laboratories without requirement of complicated instruments. The effects of formulations and process parameters of this method on the characteristics of the produced SLNs and NLCs have been investigated in several studies. This review describes the methods currently used to prepare SLNs and NLCs with focus on solvent injection method. We summarize recent development in SLNs and NLCs production using this technique. In addition, the effects of solvent injection process parameters on SLNs and NLCs characteristics are discussed.

## 1. Introduction

Solid lipid nanoparticles (SLNs) and nanostructured lipid carriers (NLCs) are colloidal particles with sizes ranging from 10 to 1000 nm [1]. They offer alternatives to other colloidal drug delivery systems, such as liposomes, emulsions, and polymeric nanoparticles. SLNs and NLCs can be prepared reproducibly in the absence of toxic organic solvents using techniques such as high-pressure homogenization or high-speed stirring [2,3,4]. The solid matrices of SLNs and NLCs contribute to increase the stabilities of active ingredients [5]. Both SLNs and NLCs are considered nanosafe carriers since they are produced from physiological and biodegradable lipids (e.g., triglycerides, partial glycerides, waxes, steroids, and fatty acids) and other materials generally recognized as safe (GRAS) [2,3,6,7]. SLNs and NLCs allow entrapment of hydrophilic and hydrophobic drugs with higher entrapment efficiencies (EEs) than liposomes [8]. Furthermore, they enable controlled drug release like polymeric nanoparticles and can be coated with appropriate ligands to target specific tissues [1]. Thus, SLNs and NLCs have been used for topical, oral, intranasal, pulmonary, and parenteral applications [9,10,11].

SLNs are considered the first generation of lipid nanoparticles with a solid matrix [12,13]. They were first developed in the mid-1990s and have attracted considerable research attention due to their combinational advantages. SLNs are composed of physiologically biocompatible excipients (like liposomes and nanoemulsions) and have solid matrices that can efficiently protect loaded drugs (like polymeric nanoparticles) [14,15]. NLCs, as the second generation, are modified from SLNs to overcome some of their drawbacks [16,17]. The major difference between SLNs and NLCs is their lipid components. SLNs are prepared from solid lipids, whereas NLCs are modified by adding liquid lipids [18,19]. The mixtures of solid and liquid lipids to produce NLCs are still solid at room and body temperature [20,21]. SLNs have low drug loadings as well as the possibility of drug expulsion during storage due to the crystallization of solid lipids. In the matrices of SLNs, drugs are located between fatty acid chains, lipid layers, and lipid imperfections. Because SLNs contain similar lipid molecules, their matrices are perfect crystals, which can be compared to a wall of bricks. Therefore, high energy modifications (*α* and *β’* forms) in SLNs matrices tend to transform to a more perfect modification (*β* form), which reduces SLNs imperfections and expels drugs [22,23]. In contrast, NLCs matrices are imperfect or amorphous structures composed of solid and liquid lipid blends [24,25]. Different lipid molecules in NLCs create more space (imperfections) for drug accommodation due to larger distances among fatty acid chains, and thus increase and better maintain drug loaded during storage [26]. The drug loadings may be enhanced by higher solubility of drugs in liquid lipids [20,27].

SLNs and NLCs have been produced using numerous methods [28,29]. Some methods, such as high-pressure homogenization, emulsion/solvent evaporation, phase inversion, and microemulsion, have been extensively investigated and reviewed [2,30,31]. Solvent injection is an alternative method for preparing SLNs and NLCs. It was first used in 1973 and has been one of effective approaches to prepare liposomes [32]. Many studies have been performed on liposome preparation using this method [33,34,35]. In 2003, Schubert et al. first used solvent injection to prepare SLNs and NLCs [36]. The advantages of this method include a fast production process and easiness of handling [37]. In addition, it can be easily performed in laboratories as no complicated instruments, such as high-pressure homogenizers or high-speed stirrers are required [38,39]. Some studies have investigated the solvent injection method for the production of SLNs and NLCs for different applications [40,41]. Others have investigated the effects of solvent injection process parameters on the physicochemical properties of SLNs and NLCs [42,43,44].

In this review, we describe the various methods used to prepare SLNs and NLCs and detail their advantages, disadvantages, and applications for drug delivery. With an emphasis on the solvent injection method, we also provide an overview of mechanisms involved and important developments made up until now using this method. Furthermore, we discuss the effects of various parameters of solvent injection method on SLNs and NLCs production and propose some suggestions regarding future developments and applications.

## 2. Methods for SLNs and NLCs Preparation

### 2.1. High-Pressure Homogenization Method

High-pressure homogenization (HPH) has been widely used to prepare nanoemulsions, SLNs, and NLCs. The technique is based on the reduction of droplet and particle size under extreme pressure conditions [45,46,47]. It provides an effective, reliable means of preparing SLNs and NLCs on a large-scale. For example, this method was used to prepare stavudine-loaded SLNs at batch sizes of up to 60 kg [48] and coenzyme Q10 loaded-NLCs at a throughput of 25 kg/h [49]. Initially, coarse lipid particles are prepared and then forced through a homogenizer with a narrow gap of few microns at high pressure [29]. The pressures (usually 100–2000 bar) accelerate the liquid mixtures to a high velocity of >1000 km/h, which generates high shear stresses and cavitational forces that reduce droplet sizes to nano dimensions [1]. This homogenization process is cycled until the desired droplet size and uniformity is obtained. The advantages of HPH are short production times, ease of production, organic solvent-free operation, and scale-up feasibility [1,49]. 

Two approaches are used to produce SLNs and NLCs using HPH, which are hot and cold homogenization. For hot HPH, drugs and molten lipids are mixed at temperatures typically 5–10 °C higher than the solid lipid melting point; drugs are either dissolved or homogeneously dispersed in the molten lipids [50]. Generally, the concentration of lipids in resultant SLNs and NLCs dispersion is 5–10% (w/v). Separately, an aqueous phase containing surfactants is preheated to the same temperature as the lipid melt, and then added under constant stirring to produce a hot pre-emulsion. The resultant pre-emulsion is homogenized using a piston-gap homogenizer at the same temperature [51]. Generally, desired SLNs and NLCs can be obtained after 3–5 homogenization cycles at 500–1500 bars. However, increasing cycle numbers and homogenization pressure may increase particle size (PS) due to particle coalescence under highly kinetic conditions [52]. After homogenization, the nanoemulsions are cooled, which results in lipid crystallization and the formations of SLNs and NLCs. This method has been used to prepare celecoxib-loaded SLNs using Precirol^®^ ATO 5 (glyceryl palmitostearate) as lipid and Cremophor^®^ EL (polyoxyl-35 castor oil) as emulsifier. These SLNs, with a PS of <200 nm and sustained drug delivery, were suggested to have potential for the treatment of breast cancer and leukemia [53]. Recently, atorvastatin-loaded SLNs were produced by HPH using Compritol^®^ 888 ATO (glyceryl behenate) as solid lipid and poloxamer 188-Phospholipon 90H as emulsifier. SLNs were investigated in a potential self-administrable eye drop formulation for the management of age-related macular degeneration. They were found to increase bioavailability in aqueous and vitreous humor and to be more stable than free atorvastatin [54]. On the other hand, hot HPH has its limitations, which include temperature-induced drug degradation and drug loss to the aqueous phase during homogenization [55]. Therefore, the technique is unsuitable for heat-sensitive or hydrophilic drugs. The temperature sensitivity issue can be overcome by cold HPH (as discussed below), whereas drug loss is usually minimized using covalently bonded lipid-drug conjugates [56]. For example, after conjugation with stearic acid and oleic acid, diminazene-loaded SLNs were prepared by hot HPH at a drug loading (DL) of 33% [57]. 

Cold HPH was developed to overcome some of the limitations of hot HPH. For cold HPH, after dissolving/dispersing drugs in molten lipids, mixtures are rapidly cooled using liquid nitrogen or dry ice. This high cooling rate provides homogeneous dispersions of drugs in lipid matrices [1]. The lipid-drug mixtures are then pulverized using a ball mill or a mortar to a PS of 50–100 µm. The lipid microparticles are suspended in cold aqueous solutions containing surfactants and then homogenized at the cold condition (e.g., 0–4 °C) usually over 5–10 cycles at 500 bars [58]. This method has been used successfully to load calf-thymus DNA and TRPsiRNA (siRNA targeting transient receptor potential vanilloid-1) into SLNs at high EEs (77.2% and 98.5%, respectively). The lipid used was a mixture of Compritol^®^ 888 ATO and stearic acid, and a Phospholipon-90H (1% *w/v*) and poloxamer-188 (2% *w/v*) mix was used as emulsifier [59]. In another study, toad venom extract was loaded into SLNs using Compritol^®^ 888 ATO as solid lipid and poloxamer 188 as emulsifier. The optimized formulation had an EE of 93% and effective anti-tumor properties [60]. Microparticle suspensions can also be prepared in ways that avoid the laborious pulverization step. For example, a solvent diffusion method was used to prepare a coarse suspension, which was subsequently subjected to cold HPH at 0–4 °C [61], which minimized drug degradation associated with hot HPH [55]. In addition, cold HPH is suitable for water-soluble drugs as it suppresses drug migration from lipid phase to the aqueous phase [1]. In another study, hot and cold HPH were compared for the preparation of SLNs loaded with didanosine (hydrophilic), and cold HPH was reported to result in higher EE (51.6% versus 32.5%) and DL (3.39% versus 0.08%) values [62]. Similar results were reported by Wang et al. [63]. In addition, several modifications can be used to improve EE and DL values further. For example, pH values of the aqueous phase can be adjusted to minimize losses of drugs with pH-dependent solubility to the aqueous phase. Using this technique, EEs of up to 90.9% have been reported [61,64]. Drug-lipid conjugates can also be used. To prepare decitabine-loaded SLNs, decitabine-stearic acid conjugates were ground into a fine powder, dispersed in a cold aqueous solution containing surfactants, and subjected to 10–14 cycles of cold HPH at a pressure of 1000 bar, at which an EE of up to 68.9% was obtained [65]. On the downside, it should be noted that cold HPH produces larger particles with a broader particle size distribution than hot HPH [1]. 

### 2.2. High-Speed Stirring and Ultra-Sonication Methods

High-speed stirring (high-shear homogenization) and ultra-sonication are widely-used dispersing techniques. High-speed stirring is one of the simplest and most cost-effective ways of producing SLNs and NLCs [55]. According to this method, lipids are first melted at high temperatures (5–10 °C higher than the melting point of solid lipids), and drugs are dissolved or dispersed homogeneously in the molten lipids. An aqueous phase containing surfactants (at the same temperature) is then added to the drug-lipid melt, and the mixture is homogeneously dispersed using a high-shear mixer. A hot oil/water (o/w) emulsion is formed due to the shear of intense turbulent eddies. SLNs and NLCs are formed by cooling these dispersions [66,67]. This high-speed stirring is usually followed by ultra-sonication, which breaks droplets based on the formation, growth, and implosive collapse of bubbles [68]. When ultra-sonication is performed without the high-shear mixing stage, SLNs and NLCs produced have a broad distribution [69], presumably because sonication energy is not transferred equally in the batch. High-speed stirring and ultra-sonication have been widely used in combination to achieve SLNs and NLCs dispersions with narrow particle distributions [70,71]. Lycopene-loaded SLNs and NLCs with PSs of <200 nm and polydispersity indices (PDIs) of 0.22–0.32 were prepared by high-speed stirring (20,000 rpm for 1 min) and subsequent ultra-sonication (~4 min). The lipids used were monostearin and medium-chain triglycerides [70]. In another study, a similar strategy was used to prepare SLNs with a low PDI value (0.215) using stearic acid as solid lipid [71]. In a study that used high-speed stirring (15,000 rpm, 10 min) followed by ultra-sonication (~3 min) to prepare linagliptin-loaded SLNs, optimized SLNs with a PS of 226 nm, a PDI of 0.18, and an EE of 73.8% were obtained, and these SLNs increased drug permeability and oral bioavailability by 3-fold as compared with linagliptin solution in rats [72]. In a recent study, high-speed stirring alone was used to prepare thiopental sodium-loaded SLNs with the objective of ameliorating obesity-induced cardiac dysfunction and hypertrophy. Compritol^®^ 888 ATO was chosen as solid lipid and Phospholipid 90 G and Tween 80 (polysorbate 80) as emulsifiers. High-speed stirring was performed at 10,000 rpm for 2–6 min followed by constant stirring at 1600 rpm for 1–4 h to produce SLNs with a PS of 68 nm and a PDI of 0.149 [73]. Both high-speed stirring and ultra-sonication are easy to handle and can be used widely without organic solvents [74]. However, both suffer from the disadvantage that drugs are exposed to high temperatures for extended times [75]. Moreover, the size distributions of SLNs and NLCs produced by high-speed stirring are broad and particles are micro-sized [3]. The disadvantage of ultra-sonication is that products are contaminated with metals originating from sonicator probes [1]. Bath sonication provides a means of circumventing this problem but must be combined with other follow-on procedures to reduce PS and PDI values [76]. Furthermore, using high-speed stirring and/or ultra-sonication methods require high surfactant, whereas total lipid concentrations are low [3].

### 2.3. Microemulsion Method

This method was first developed in the early 1990s [77] and has since been investigated by several researchers [75,78]. The method involves diluting a microemulsion in a cold aqueous solution, which results in the formation of nanoemulsion and the subsequent formation of SLNs and NLCs by lipid precipitation. Briefly, a drug is dissolved in molten lipids at a temperature above the lipids melting point, and then an aqueous phase containing water and surfactant (pre-heated to the same temperature) is added under mild stirring to form a transparent and thermodynamically stable microemulsion [79,80]. The microemulsion is then poured into a cold aqueous solution (2–10 °C) under gentle mechanical mixing [55]. Typically, the volume of the cold aqueous phase is 25 to 50 times greater than that of the hot emulsion [1]. Upon dilution, a nanoemulsion is formed and lipids immediately crystallize to form SLNs or NLCs [81]. This method was successfully applied to prepare curcumin loaded SLNs with small PSs (< 200 nm) and low PDIs (< 0.2). The SLNs were more effective than free curcumin (5–10-fold) on P-glycoprotein mediated doxorubicin-resistant triple-negative breast cancer cells [82]. Recently, the microemulsion method was used to prepare SLNs loaded with isotretinoin and α-tocopherol acetate using monostearin as solid lipid and polysorbate 80 as emulsifier. SLNs showed a small PS (193 nm) and high EEs (84% for isotretinoin and 77% for α-tocopherol acetate). SLNs gel was subsequently prepared using Kolliphor-407 P as a gelling agent. It showed sustain-release of drugs for 24 h in vitro and potent efficacy in a rat model of acne [83]. The microemulsion method is simple and reproducible, and can be scaled up. For large-scale production, the microemulsion can be prepared in a large temperature-controlled tank and pumped into another tank containing cold water for lipid precipitation [55]. In addition, this method is solvent-free. Its limitations are the large amount of water required to dilute microemulsions and high surfactant usage [84]. Excess water can be removed by lyophilization or ultra-filtration to concentrate SLNs and NLCs dispersions [55].

### 2.4. Solvent Emulsification-Diffusion Method

The solvent emulsification-diffusion method is mainly used to produce polymeric nano-carriers. In 2003, Trotta et al. first used this technique to prepare SLNs and NLCs [85]. This method is generally performed using organic solvents that are partially miscible with water, such as methyl acetate, ethyl acetate, isopropyl acetate, benzyl alcohol, and butyl lactate. Initially, the organic solvent and water are mutually saturated with each other to obtain the initial thermodynamic equilibrium of both phases. Lipids and drugs are dissolved in the water-saturated solvent, which is then emulsified in the aqueous phase (solvent-saturated water containing stabilizer) under stirring to form an o/w emulsion. The emulsion is diluted with water (volume ratio from 1:5 to 1:10) to allow diffusion of the solvent into the continuous phase. SLNs and NLCs are formed spontaneously due to lipid precipitation and the solvent is then eliminated by lyophilization or vacuum distillation [85,86]. Tamoxifen-loaded SLNs were prepared using this method. The lipid (α-lipoic acid-stearylamine conjugate) and tamoxifen were dissolved in a water-saturated solvent and subjected to high-speed homogenization in a solvent-saturated aqueous solution containing 2% *w/v* stabilizer. The obtained emulsion was then mixed with water to form SLNs, and the organic solvent was removed using a rotary evaporator under reduced pressure. Of three solvents screened (ethyl acetate, n-propyl acetate, and isopropyl acetate), SLNs prepared with isopropylacetate were the optimal formulation with a PS of 233 nm and a PDI of 0.114 [87]. In a recent study, letrozole-loaded SLNs were prepared using this method, although mutual saturation of solvent and water was not required. The solid lipid (tripalmitin glyceride) and letrozole were dissolved in ethanol at 75–80 °C, and this solution was emulsified in hot water containing 3% polysorbate 80. Finally, the ethanol was evaporated by stirring to yield SLNs with a PS of 228 nm and a PDI of 0.349 [88]. This method can avoid exposing drugs to high temperatures and physical stress like those associated with high-speed stirring or high-pressure homogenization. In addition, it can be easily scaled-up [89]. Furthermore, the technique can be applied to hydrophilic and hydrophobic drugs. Different drugs and biomolecules, such as insulin [90,91], tretinoin [11], and cyclosporine [92] have been encapsulated in SLNs and NLCs using this technique. However, like the microemulsion method, the solvent emulsification-diffusion method is associated with substantial dilution of SLNs and NLCs dispersions and requires a purification process to remove residual organic solvent [55,85].

### 2.5. Solvent Emulsification-Evaporation Method

Unlike the emulsification-diffusion method, water-immiscible organic solvent (e.g., chloroform, cyclohexane, dichloromethane, and toluene) are used to prepare SLNs and NLCs using the solvent emulsification-evaporation method [93,94]. Briefly, drug and lipids are dissolved in a solvent or a solvent mix, and then emulsified in an aqueous phase to form nanodispersions. Thereafter, the organic solvent is evaporated by mechanical stirring or in a rotary evaporator. SLNs and NLCs are formed due to lipid precipitation after solvent evaporation [95,96]. Rifampicin-loaded SLNs were prepared using this method. Monostearin, soya lecithin, and rifampicin were dissolved in chloroform and then emulsified in an aqueous phase containing 1.5% polysorbate 80 by high-speed homogenization and ultrasonication. Solvent evaporation was achieved by stirring. The drug-loaded SLNs had a PS of 49 nm, a PDI of 0.31, and an EE of 68.7% [94]. Similarly, to prepare cefuroxime axetil-loaded SLNs, stearic acid, tristearin, soya lecithin, and the drug were dissolved in an organic mixture of chloroform and dichloromethane (3:1). The organic phase was then heated to 70 °C and added to an aqueous phase (poloxamer 188, preheated to 70 °C) under high-speed homogenization and ultrasonication. After SLNs formed, a rotary evaporator was used to remove organic solvents under reduced pressure [97]. The concentration of lipids in the organic phase has a considerable effect on mean PSs of SLNs and NLCs prepared using the solvent emulsification-evaporation method. Small SLNs and NLCs are obtained at a low lipid concentration [1]. The main advantage of this method is avoidance of drug exposure to high temperatures, and thus, it is useful for encapsulating highly thermo-labile drugs. Furthermore, SLNs and NLCs produced using this method and the solvent emulsification-diffusion method have PSs of around 100 nm and narrow size distributions [3]. The limitations of the solvent emulsification-evaporation method are that it requires toxic organic solvents and the suspension produced is dilute and requires further evaporation or ultra-filtration [75]. Both the solvent emulsification-diffusion and solvent emulsification-evaporation methods involve the use of organic solvents, which means additional solvent removal steps are needed. Furthermore, in vitro and in vivo risk assessments on the SLNs and NLCs produced are also required. The residual solvent levels should be below specified values, at which no adverse effect is observed [98].

### 2.6. Double Emulsion Method

The double emulsion method provides a means of producing SLNs and NLCs of hydrophilic drugs and biomolecules (e.g., peptides and proteins) [99,100,101]. Particularly, it has been widely used to incorporate insulin into the matrices of SLNs and NLCs for oral delivery [102,103,104]. According to this method, a drug and a stabilizer are dissolved in an aqueous solution and then emulsified in a water-immiscible organic phase containing lipids [105] or in solvent-free molten lipids [106,107]. These primary emulsions are dispersed in an aqueous phase containing a hydrophilic emulsifier to form water/oil/water (w/o/w) emulsion. After solvent evaporation, SLNs and NLCs dispersions are obtained due to lipid precipitation [108]. Notably, for this method, the stabilizer essentially prevents drug partitioning to the external water phase during the solvent evaporation. The double emulsion method has been used to prepare raloxifene-loaded SLNs as follows. Monostearin and lecithin were first dissolved in chloroform, and raloxifene was dissolved in a methanol:water mixture, which was then emulsified in the chloroform solution to form a water/oil (w/o) emulsion. This emulsion was injected into an aqueous poloxamer 188 solution under high shear to form a w/o/w emulsion. Organic solvents were evaporated using a rotary evaporator to form SLNs, which were harvested by centrifugation (45,000 × *g*, 1 h). The obtained SLNs had a PS of 180 nm and an EE of 4.5% and exhibited greater cytotoxic effects on MCF-7 cells than free raloxifene [105]. In another study, instead of using solvents, the lipids (stearic acid and capric/caprylic acid) were melted. A model compound (sulforhodamine 101 or coumarin-6) was dissolved in hot water and emulsified in the molten lipids to form w/o emulsion. After w/o/w emulsion was formed, the emulsion was poured into cold water (2–5 °C) to solidify NLCs [107]. A similar strategy was used to prepare diethyldithiocarbamate-loaded SLNs with PSs of <200 nm and EEs of >80% [106]. The advantage of the double emulsion method is that it does not require molten lipid. However, EE and DL values of SLNs and NLCs are low due to leaching of the hydrophilic drugs to the outer aqueous phase. EEs of SLNs and NLCs produced using this technique may range from 5.2–40.3% for insulin [108] or relatively higher (63%) for sulforhodamine 101 [107]. In some cases, EEs can also reach 80% for diethyldithiocarbamate [106] and 90% for polymyxin B sulfate [109]. In addition, the SLNs and NLCs prepared using this method have relatively large PSs (up to micro sizes) [55,75].

### 2.7. Phase Inversion Temperature (PIT) Method

The PIT method has been used to produce nanoemulsions, SLNs, and NLCs [110,111,112]. The technique is based on the temperature-induced inversions of w/o to o/w emulsions and vice versa. The method requires the use of non-ionic polyoxyethylated surfactants with temperature-dependent properties. The hydrophilic-lipophilic balance (HLB) value of these surfactants is high at low temperatures because their hydrophilic groups are highly hydrated. When temperature is increased, dehydration of ethoxy group occurs, which increases surfactant lipophilicity and decreases HLB values of the surfactants [111]. PIT is defined as the temperature at which the affinities of the surfactants for aqueous and lipid phases are equal [113,114]. At temperatures > PIT, the surfactants favor the formation of w/o emulsions, whereas at temperatures < PIT, they turn to form o/w emulsions [115]. To produce SLNs and NLCs, oil, water, and surfactant are first heated to a temperature > PIT under stirring to form w/o emulsions. Subsequently, they are rapidly cooled with continuous stirring, which promotes the breakdown of w/o microemulsions and induces the formation of o/w nanoemulsions. SLNs and NLCs are formed when lipids are precipitated at low temperatures [116]. This method was used to prepare metronidazole-loaded NLCs from monostearin and Capmul MCM (glyceryl monocaprylate). NLCs had a PS of < 300 nm and an EE of ~40%. NLCs gel containing Carbopol 974 P increased skin retention and antibacterial activity of the drug as well as provided drug sustained-release [117]. SLNs and NLCs prepared using this method have been reported to have small PSs, narrow size distribution, and excellent stabilities [118,119]. For example, NLCs loaded with β-carotene and α-tocopherol had a PS of 35 nm [120]. This method requires little energy input and does not require organic solvents. However, it involves low stabilities of the nanoemulsion formed, and sometimes requires several temperature cycles (e.g., three cycles between 60 and 90 °C) [121,122].

### 2.8. Membrane Contactor Method

In this method, a specific membrane contactor is used for producing SLNs and NLCs. Briefly, a lipid phase is pressed through the pores of a membrane while temperature is maintained above the solid lipid’s melting point. This step results in the formation of small droplets. At the same time, an aqueous phase containing surfactants circulates over the other side of the membrane inside a module. It flows tangentially to the membrane surface and sweeps away droplets formed at pore outlets. SLNs and NLCs are formed by allowing the hot emulsion to cool down to room temperature [123]. The effects of formulation and process parameters, including the membrane pore size, lipid phase pressure, flow velocity of the aqueous phase, and temperatures of the aqueous and lipid phases on SLNs and NLCs size are intensively investigated. PS can be adjusted by changing lipid phase flux through membranes. For example, SLNs with PSs of 175–260 nm were produced using a lipid phase flux between 0.21 and 0.27 m^3^/h·m^2^ [124,125]. This method has been used to prepare vitamin E-loaded liposomes, micelles, nanoemulsions, and SLNs for pulmonary drug delivery [126]. This method is feasible for scaling-up as a membrane with a pore size of 0.1 μm resulted in high fluxes [124,127]. However, the limitations of this method are that it requires a sophisticated system and membranes are prone to clogging.

### 2.9. Supercritical Fluid-Based Methods

Several SLNs and NLCs production methods involved the use of supercritical fluids like supercritical CO_2_. For the supercritical fluid extraction of emulsions (SFEE) method, an o/w emulsion is prepared beforehand, followed by supercritical fluid extraction of the organic solvent. Typically, the emulsion is added to an extraction column from the top, and supercritical CO_2_ is introduced in a counter-current manner from the bottom. SFEE has a much higher solvent extraction efficiency than other methods that use evaporation, diffusion, and dilution. The solvent is quickly and completely removed, and this leads to lipid precipitation. Furthermore, the produced SLNs and NLCs have uniform particle size distribution [128,129]. The o/w emulsions are prepared using water-immiscible solvents [128] and water-partially miscible solvents [130]. This method was used to prepare SLNs loaded with indomethacin and ketoprofen. Emulsions were prepared by dissolving a lipid (tripalmitin, tristearin, or Gelucire^®^ 50/13) and a model drug in chloroform, dispersing the organic solution into an aqueous phase of sodium glycocholate, and passing the mixture through a high-pressure homogenizer. The o/w emulsion was then introduced into the top of an extraction column (4 L volume, 1 m long) at a constant flow rate of 2 mL/min. Supercritical CO_2_ was introduced simultaneously at a flow rate of 40 g/min. The produced SLNs had PSs of <30 nm and DLs of 10–20% [128]. Similarly, praziquantel-loaded SLNs were prepared using this method. Cetyl palmitate, praziquantel, polysorbate 80, and soya lecithin were solubilized in dichloromethane and then emulsified in an aqueous phase. The emulsion was introduced into the top of a continuous extraction column, whereas supercritical CO_2_ was from the bottom. Batch sizes ranged from 100 to 5000 mL. The resultant SLNs had a PS of 25 nm, a PDI of 0.5, and an EE of 88.4% [129]. 

Supercritical CO_2_ is also used in a method called supercritical assisted injection in a liquid antisolvent (SAILA). This method is similar to the solvent injection method, which is discussed below. In brief, lipids and drugs are dissolved in a water-miscible organic solvent, mixed with supercritical CO_2_ and injected into an aqueous phase containing surfactant. The mixing of the two fluids results in a rapid supersaturation and consequent precipitation of SLNs or NLCs [131]. Trucillo et al. used this method to prepare SLNs from stearic acid, soya lecithin, or cholesterol with PSs ranging from 158 to 462 nm [131]. In another method, an organic solvent containing lipids and a drug was mixed with supercritical CO_2_ and then expanded through a nozzle, which led to the formation of SLNs due to rapid CO_2_ evaporation. This method was successfully used to prepare camptothecin-loaded SLNs based on Compritol^®^ 888 ATO that increased the cytotoxic effects of camptothecin on MCF7 and MCF10A cells [132]. Supercritical fluid-based methods usually result in uniform particle size distributions and their solvent extraction efficiencies are higher than those of conventional extraction methods. However, they are expensive and involve the use of organic solvents [130].

### 2.10. Coacervation Method

Coacervation has been widely used to produce polymeric nanoparticles [133,134] and was first used by Battaglia et al. to prepare SLNs in 2010 [135]. This method is based on the acidification-induced precipitation of the alkaline salts of fatty acids. The lipids used are in alkaline salt forms (e.g., sodium stearate), which are dispersed in an aqueous solution of a polymeric stabilizer, such as PVA or HMPC [136]. Drugs can be dissolved in the lipid phase [137] or loaded into blank SLNs in later steps [138]. To load drugs into the lipid phase, drugs are solubilized in ethanol and dissolved in the lipid. The mixture is heated to a temperature above its Krafft point, when a clear micellar solution of the lipid alkaline salts is formed. An acidifying solution (coacervating solution) is then added dropwise to this solution, which causes the lipids to precipitate. The obtained suspension is then cooled in a water bath with stirring at 300 rpm until a temperature of 15 °C is reached then cooled in a water bath with stirring to complete the precipitation of SLNs or NLCs [139]. This method was used to prepare SLNs and NLCs loaded with insulin or glargine insulin. Sodium stearate was used as fatty acid salt, PVA 9000 as stabilizer, and 1M lactic acid as coacervating solution. Glargine insulin-loaded NLCs exhibited effective uptake ex vivo and achieved an estimated absolute bioavailability of 6% in blood after oral administration in rats. In addition, this formation also reduced blood glucose levels in healthy rats [140]. In a recent study, temozolomide-loaded SLNs were prepared from sodium behenate and PVA 9000. The optimized SLNs exerted greater anti-proliferative effects on melanoma cells, and in mice, inhibited the growth and vascularization of B16-F10 melanoma without discernible toxic effects [138]. The coacervation method provides a simple solvent-free means of preparing SLNs and NLCs without sophisticated instruments. However, it can only be used on lipids that form alkaline salts, such as fatty acids, and is not suitable for pH-sensitive drugs [139].

### 2.11. Solvent Injection Method

**Figure 1:**
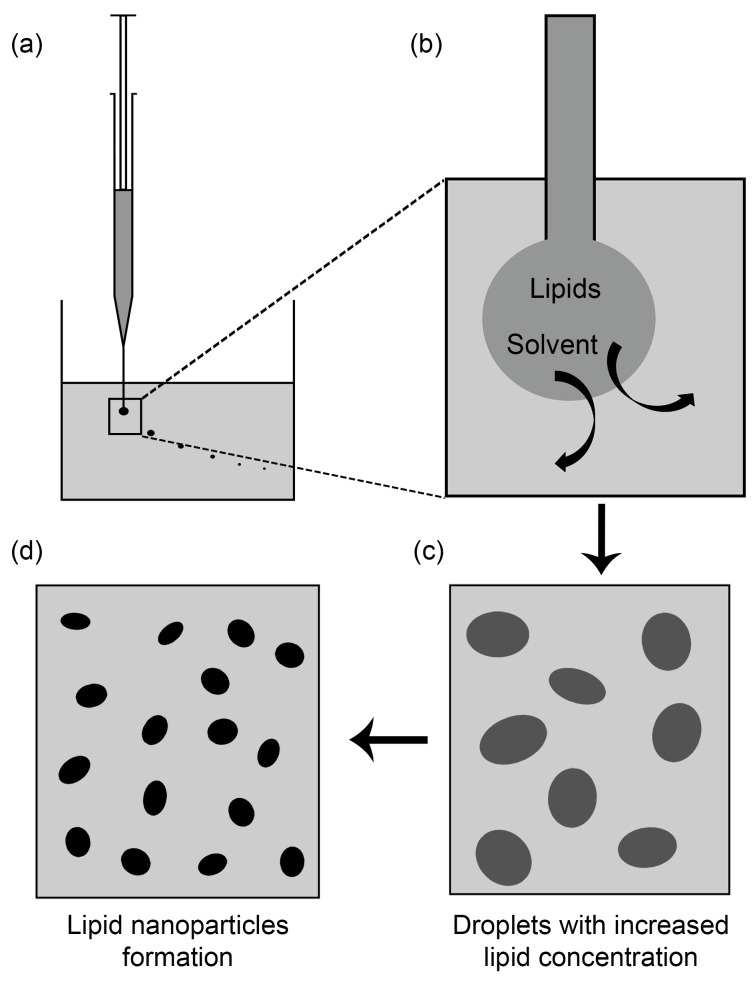
Schematic representation of lipid-nanoparticle formation using the solvent injection method. Lipids and drugs are dissolved in a water-miscible solvent (organic phase) and injected into an aqueous phase containing emulsifiers (**a**). Following injection, the solvent gradually diffuses into the aqueous phase (**b**), which leads to droplet division and a reduction in droplet size while lipid concentration is increased (**c**). Consequently, solid lipid nanoparticles and nanostructured lipid carriers are formed and stabilized by the emulsifiers (**d**).

The solvent injection method used for preparing SLNs and NLCs was first reported by Schubert et al. in 2003 [36]. According to this method, lipids and drugs are dissolved in a water-miscible solvent (e.g., methanol, ethanol, isopropanol, or acetone) or a water-miscible solvent mixture. The aqueous phase is usually prepared by adding an emulsifier or an emulsifier mixture to water or a buffer solution. The organic phase is then quickly injected into the aqueous phase under continuous mechanical stirring using a needle [55]. The basic principles of this method and the solvent emulsification-diffusion method are similar. A schematic representation of SLNs and NLCs formation using the solvent injection method is illustrated in Figure 1. Following injection, two principal mechanisms occur simultaneously and aid each other to form SLNs and NLC. First, the solvent diffuses out of the droplets into the aqueous phase, which results in a droplet size reduction. As a consequence, lipid concentration within the droplets increases, which leads to the formation of local supersaturated regions stabilized by emulsifiers in the aqueous phase [36]. Second, the emulsifiers reduce interfacial tension between water and solvent, and this leads to formations of small solvent-lipid droplets at the injection site. Due to the interfacial pulsation and turbulence during solvent diffusion, those droplets are broken into smaller droplets with essentially the same lipid concentrations [36,76]. The free energy released when the solvent is redistributed to its equilibrium state provides the energy required for droplet division [36]. Therefore, in the solvent injection method, solvent diffusion results in the formation of tiny droplets and lipid precipitation. Emulsifiers play an important role in the determination of PSs and size distributions. When present, even at low concentrations (e.g., 0.1%), PSs and are markedly reduced [36,76]. In general, as the concentration of emulsifiers increases, initially PS and PDI decrease, but when its concentration reaches a critical value (0.5–1.5%), PS and PDI start to increase [76]. This issue is discussed in detail in Section 5. Consequently, SLNs and NLCs are formed and stabilized by the emulsifier. Notably, the diffusion rate of organic solvent into the aqueous phase is considered to be one of the most critical factors affecting PSs and size distributions [36,141]. 

This method has been modified in some studies, including a micro-channel with a cross-shaped junction [142] or a co-flowing micro-channel system [143]. The micro-channel with a cross-shaped junction consists of a microchannel module, two precision syringe pumps for supplying an aqueous phase and a solution of lipid in solvent, a digital inversion microscope, and a stirred unit to collect the SLNs suspension. The process was carried out as follows: an organic solution (Softisan 100 in acetone, viscosity 0.35 mPa) was fed into the main microchannel, and an aqueous phase (0.5% poloxamer 188, viscosity 1.46 mPa) was pumped into the inlets of the two branch channels at an equal flow rate. A digital inversion microscope system was used to capture images of flow field behaviors in microchannels. The produced SLNs had PSs of 100–200 nm [142]. In the co-flowing micro-channel system, two precision syringe pumps were also used to supply aqueous and lipid-solvent phase. The inner capillary had inner and external diameters of 110 and 490 μm, respectively, whereas these values of the outer capillary were 650 and 7000 μm, respectively. An organic solution (Softisan 100 in acetone) was fed into the inner capillary, and simultaneously, an aqueous phase (poloxamer 188) was pumped into the outer capillary at the same flow rate. The produced SLNs had small PSs (<250 nm) and narrow size distribution (PDIs <0.26) [143]. Another modification of solvent injection method is the microfluidic rapid ethanol dilution method using baffle devices. This method was used as a post-treatment and provided precise size control of SLNs and NLCs prepared using the solvent injection method. For the SLNs and NLCs preparation step, 1-palmitoyl-2-oleoyl-sn-glycero-3-phosphocholine (POPC) in ethanol and saline were introduced into the baffle device. Post-treatment was performed on-device and involved diluting the SLNs and NLCs suspensions (with 25% *v/v* ethanol) to 1%. This post-treatment was applied to prepare siRNA-loaded NLCs with a small PS (33 nm) and a high EE (>90%). The formulation specifically knocked down the plasma coagulation factor VII [144]. Other applications of the solvent injection method are discussed in Section 3. Table 1 summarizes the mechanisms, advantages, and disadvantages of different SLNs and NLCs preparation methods.

## 3. Preparation of SLNs and NLCs Using Solvent Injection Method in Recent Studies

A number of studies have used the solvent injection method to produce SLNs and NLCs, and various drugs have been loaded into these carriers for different applications. Here, we present major SLNs and NLCs systems and details of applications to date that use this method. 

### 3.1. Oral Delivery

SLNs and NLCs have been widely investigated for oral delivery. Several studies have reported the successful use of SLNs and NLCs for liver targeting. Singh et al. performed a Box-Behnken design to optimize thymoquinone-loaded SLNs at a PS of 166.1 ± 10.96 nm and an EE of 71.60 ± 3.85%. Oral bioavailability in rats of the optimized formulation was nearly 5-fold that of the drug suspension [145]. The same group later loaded resveratrol into SLNs, which were optimized at a PS of 191.1 ± 10.44 nm, a PDI of 0.156 ± 0.05, and an EE of 73.7% using the solvent injection method. The solvent used was ethanol, and the lipids used were Compritol^®^ 888 ATO and Gelucire^®^ 50/13. Drug release in vitro was extended to 24 h, and in an in vivo study in rats, these SLNs had a 5-fold increase in oral bioavailability as compared with a resveratrol suspension [146]. A pharmacodynamic evaluation was conducted in both of these studies. Compared with control and marketed (Silybon VR) formulations, the optimized SLNs induced significant decreases in serum biomarker enzymes (serum glutamic oxalo-acetic transaminase (SGOT), serum glutamic pyruvic transaminase (SGPT) and alkaline phosphatase (ALP)) after oral administration in rats with paracetamol-induced liver cirrhosis [145,146]. Histopathologic examination of liver microtome sections confirmed the effects of treatment in both studies [145,146]. Finally, liver mRNA from animals treated with the optimized formulation showed significant down-regulations of tissue inhibitor of metalloproteinases-1 and nuclear factor-kB [146]. Another study reported the preparation and application of paclitaxel-loaded SLNs. Stearylamine was used as solid lipid, and soya lecithin and poloxamer 188 as emulsifiers. The SLNs had a small PS of 96 ± 4.4 nm, a PDI of 0.162 ± 0.04, an EE of 75.42 ± 1.5%, and a DL of 31.5 ± 2.1%. After oral administration of paclitaxel-loaded SLNs, drug levels in plasma and tissues were 10- and 2-fold higher, respectively, as compared with the free drug solution. Absorbed drug was distributed in liver, lungs, kidneys, spleen, and brain. Furthermore, toxicity studies confirmed the relatively safe nature of SLNs, which caused no significant changes in total leucocyte count, differential leucocyte count, or in serum lactate dehydrogenase (LDH), SGOT, and SGPT levels [147]. Simvastatin-loaded NLCs were prepared using glycerol monostearate and oleic acid as lipids, poloxamer 407 as emulsifier, and isopropanol as solvent. A 2^3^ factorial design was adopted to optimize NLCs at a PS of 212 nm, a PDI of 0.344, and an EE of 84%. The optimized formulation achieved an increase in bioavailability of 4.8- and 2.3-fold as compared with simvastatin suspension and simvastatin-loaded SLNs, respectively after oral administration in mice. In addition, a biodistribution study demonstrated preferential NLCs accumulation in liver, the target organ for simvastatin [42]. In another study, asiatic acid-loaded SLNs were prepared using monostearin as lipid and poloxamer 188 as surfactant. A Box-Behnken design was used to obtain the optimized formulation, which provided a PS of 237 nm and an EE% of 64.4%. Oral bioavailability in rats of drug-loaded SLNs was 2.5-fold higher than that of the drug alone [148]. Adefovir dipivoxil, a nucleoside reverse transcriptase inhibitor, was loaded into SLNs using the solvent injection method. SLNs had a PS of 267 ± 18 nm and an EE of 73.5 ± 2.12%. In rats, the relative bioavailability of adefovir-loaded SLNs was 78.23%, higher than those values of nanosuspension (52.46%) and micro-suspension (34.34%) after oral administration [149].

Several studies have reported anti-cancer applications of SLNs and NLCs. Parveen et al. prepared andrographolide-loaded SLNs using the solvent injection method. The lipid was cetyl alcohol, the emulsifier was polysorbate 80, and the solvent was ethanol. The optimized SLNs had a small PS of 154 nm, a PDI of 0.172, a high EE of 91.4%, and a high DL of 18.6%. SLNs increased oral bioavailability (3.41-fold) and antitumor activity as compared with the drug suspension in mice. Antitumor activity was assessed using mean survival times, percentage increases in life spans, and tumor growths [150]. In another study, Hashem et al. prepared tamoxifen-loaded SLNs using monostearin or stearic acid as solid lipid, polysorbate 80 or poloxamer 188 as emulsifier, and methanol as solvent. The optimized formulation had a PS of 130 nm, a PDI of 0.231, and an EE of 86.1%, and showed antitumor activity similar to the free drug against MCF-7 cells (a human breast cancer cell line). Oral bioavailability in rats was ~1.6-fold greater than that of the free drug [151]. 

Brain targeting via oral delivery is also a potential application of SLNs and NLCs. A 3^2^ full factorial design was used to optimize sumatriptan succinate-loaded SLNs prepared using the solvent injection method. SLNs were coated with chitosan to improve brain distribution via oral delivery. PSs and EEs of all batches fell in the ranges 192–301.4 nm and 76.3–91.1%, respectively. Two hours after oral drug administration in rats, the optimized formulation exhibited a 4.5-fold increase in the brain/blood ratio of the drug. The optimized formulation was suggested to provide an effective approach to the therapeutic management of migraine as it successfully targeted the brain following oral administration [152]. In another study, puerarin-loaded SLNs were prepared using monostearin as solid lipid, soya lecithin and poloxamer 188 as emulsifiers, and a methanol:ethanol mixture as solvent. The SLNs enhanced the oral bioavailability of puerarin by ~3-fold in rats. In addition, tissue concentrations of the drug increased, particularly in heart and brain [39]. Recently, a 4^2^ full factorial design study was used to optimize temazepam-loaded NLCs prepared by solvent injection method. The optimum formulation consisted of Compritol^®^ 888 ATO:oleic acid (3:1) as lipids and poloxamer 407 as emulsifier and achieved a PS of 306.6 ± 49.6 nm, a PDI of 0.09 ± 0.10, and an EE of 75.2 ± 0.1%. Scintigraphy images showed an evident increase in brain uptake for an oral ^99m^Tc-temazepam-loaded NLCs formulation versus a ^99m^Tc-temazepam suspension, whereas the oral bioavailability of the ^99m^Tc-temazepam-loaded NLCs was ~3-fold higher. In addition, a brain distribution study demonstrated brain targeting by the drug-loaded NLCs (3.4-fold increase) in rats [153].

### 3.2. Parenteral Delivery

SLNs and NLCs may be injected intravenously to target particular organs. Particles are cleared from the circulation by the liver and spleen. A reticuloendothelial system avoidance (stealth) facility may be incorporated to facilitate drug targeting (e.g., in tumor tissues). It can be conducted using polyoxyethylene [3]. SLNs and NLCs produced by solvent injection method have been investigated for parenteral application in various studies. Some studies aimed to enhance anticancer activity. For example, doxorubicin hydrochloride-loaded SLNs were prepared using the solvent injection method and then mannosylated. Mannosylated SLNs had a PS of 360 nm, a PDI of 0.135, and an EE of 70.3%. Following intravenous injection via tail veins in mice, these mannosylated SLNs showed drug sustained-release for 48 h. Furthermore, as compared with the free drug, bioavailability was around 3- and 5-fold higher for SLNs and mannosylated SLNs, respectively. Mannosylated SLNs also achieved higher drug concentrations in the tumor mass [38]. In a later study on doxorubicin-loaded SLNs produced using vitamin B6-stearic acid conjugate as solid lipid, SLNs enhanced doxorubicin release at pH 5, which was attributed to the pKa (5.6) of vitamin B6. Following intravenous injection via tail veins in rats, pH-sensitive SLNs significantly prolonged doxorubicin blood circulation time, increased drug accumulations at tumor sites, and enhanced therapeutic efficacy. They were also less toxic in tumor-bearing rats than the free drug. The lower toxicity of the formulation was further confirmed by histological and survival analyses [154]. In another study, paclitaxel-loaded SLNs were prepared using tristearin and distearoyl-phosphatidyl ethanolamine as lipids, polysorbate 80 as emulsifier, and an acetone:ethanol mixture as solvent. Drug-loaded SLNs were mannosylated and had a PS of 254 nm and a PDI of 0.312. After intravenous injection in rats, mannosylated SLNs were more cytotoxic to A549 cells and caused higher drug concentrations in alveolar cell sites than drug-loaded SLNs [155].

Other studies have reported various applications of SLNs and NLCs prepared using the solvent injection method. Aceclofenac-loaded SLNs were prepared beforehand and then conjugated with chondroitin sulfate (CS-SLNs). These CS-SLNs had a PS of 154.2 ± 1.1 nm, a PDI of 0.403, and an EE of 65.4 ± 1.7%. After intravenous administration in rats, SLNs and CS-SLNs increased aceclofenac levels in inflammatory knee joints (by 2- and 10-fold versus drug solution, respectively). In addition, CS-SLNs inhibited edema for up to 12 h, whereas SLNs and aceclofenac solution inhibited it for 8 and 4 h, respectively [156]. In another study, rifampicin-loaded SLNs were conjugated with lactoferrin to enhance SLNs delivery to lungs. The optimized formulation had a PS of 271 nm, a PDI of 0.124, and an EE of 68.4%. Lactoferrin-coupled SLNs increased drug uptakes by lungs (47.7%, by ~3-fold compared with uncoupled SLNs) following IV injection in rats. A fluorescence study confirmed enhanced uptake of lactoferrin-coupled SLNs in lungs [157]. To enhance drug delivery to brain for the treatment of HIV-1 associated dementia, nifedipine-loaded SLNs were prepared and coated with Tween 80. The lipids used were tristearin and hydrogenated soya phosphatidylcholine (1.5:1 w/w). The produced SLNs had a small PS of 120 nm and an EE of 70%. Tween 80-coated SLNs increased percentage DNA fragmentation in vitro, cell viability, and drug accumulation in brain as compared with nifedipine alone and uncoated SLNs [158]. In a recent study, siRNA-loaded NLCs were prepared and post-treated using an integrated baffle device. The NLCs had a small PS (33 nm) and a high EE (90%). Following intravenous injection in mice, siFVII knocked down the plasma coagulation factor VII levels in liver tissue by >80%. Furthermore, NLCs were specifically delivered to extravascular regions with low accumulation around blood vessels [144].

In addition to intravenous administration, several studies have produced SLNs and NLCs for subcutaneous injection. For example, ondansetron hydrochloride-loaded NLCs were prepared using tripalmitin and Phosal^®^ 53MCT as lipids, polysorbate 80 as emulsifier, and ethanol as solvent. The optimized formulation obtained after screening for various process parameters had a small PS (185 nm), a low PDI (0.214), a high EE (93.2%), and a high DL (10.43%). Following subcutaneous administration in rats, the NLCs showed sustained-release for up to 96 h. The formulation was suggested to have potential for the treatment of patients suffering from chemotherapy-induced nausea and vomiting by reducing dosing frequency and increasing patient compliance [76]. In another report, hepatitis B surface antigen (HBsAg) was loaded onto the surfaces of SLNs for vaccine delivery against hepatitis B and delivered subcutaneously. These SLNs were prepared using tristearin as solid lipid, polysorbate 80 as emulsifier, and acetone as solvent using the solvent injection method. After mannosylation, the SLNs had a PS of 96.6 nm, a PDI of 0.05, and an EE of 64.5%. These mannosylated SLNs increased cellular uptake, reduced toxicity, and increased immune response. In addition, they produced sustained antibody titers, indicating better immunological potential [159].

### 3.3. Topical Delivery

Various authors have used SLNs and NLCs for the topical delivery of different drugs. Miconazole is an antifungal used to treat ringworm, pityriasis versicolor, and yeast infections of skin. Miconazole nitrate-loaded SLNs were prepared from tristearin, soya lecithin, and polysorbate 80 using ethanol as solvent. Various process and formulation parameters were varied to optimize SLNs at a PS of 206 nm, a PDI of 0.21, and an EE of 90.9%. The SLNs were loaded into a hydrogel for topical application. The gel increased skin retention 10-fold as compared with miconazole suspension and miconazole hydrogel. It was also more efficient at treating candidiasis in rats [160]. Terbinafine is an antifungal used to treat pityriasis versicolor, fungal nail infections, and ringworm. SLNs of terbinafine hydrochloride were prepared using the solvent injection method. The lipid used was Compritol^®^ 888 ATO, the emulsifier was poloxamer 407, and the solvent was isopropanol. A 3^3^ factorial design was used to obtain optimized SLNs with a PS of 274 nm, a PDI of 0.32, and an EE of 74.6%. The SLNs gel increased skin retention ex vivo through rat abdominal skin and reduced the fungal burden of *Candida albicans* more than a commercial product in rat pharmacodynamics studies [44].

Adapalene is a topical retinoid primarily used to treat mild-moderate acne, keratosis pilaris, and other skin conditions. SLNs loaded with this drug were prepared from tristearin, soya lecithin, polysorbate 80 using an acetone:ethanol mixture as solvent. The optimized SLNs had a PS of 148 nm, a PDI of 0.169, and an EE of 89.9%. They were then loaded into carbopol hydrogel. This gel showed sustained-release in vitro for 48 h using rat skin. In addition, an accumulation of SLNs was observed, particularly in epidermis [161]. Halobetasol propionate is used to treat various skin conditions, such as eczema, dermatitis, psoriasis, and rashes. SLNs loaded with this drug were prepared using monostearin as solid lipid, polysorbate 80 as emulsifier, and isopropanol as solvent. A 3^2^ full factorial design was applied to obtain optimized SLNs, which had a PS of 200 nm and an EE of 93%. SLNs-based carbopol gels were produced and exhibited drug sustained-release (up to 12 h) on human cadaver skin. They reduced systemic uptake, increased drug accumulation in skin, and were nonirritant to rabbit skin [162]. Mometasone furoate is a steroid usually used to treat fever, asthma, and certain skin conditions. To overcome the shortcomings of conventional formulations, SLNs loaded with mometasone furoate were prepared using the solvent injection method. The optimized SLN-based gel showed a 15.2-fold increase in skin permeability as compared with a commercial cream. Its deposition in skin was 83.52%, which was 2.67-and 20-fold higher than a commercial cream and the plain drug-loaded gel, respectively [163].

### 3.4. Nose-to-Brain Delivery

Nose-to-brain delivery allows the transportation of drugs to the central nervous system following administration to the roof of the nasal cavity. Drugs can bypass the blood-brain barrier since they access the brain directly from the nasal cavity [164]. Ondansetron hydrochloride, a drug used to treat chemotherapy-induced nausea and vomiting in cancer patients, was loaded into SLNs. The lipid used was monostearin, the emulsifier was a lecithin:poloxamer 188 mixture, and the solvent was ethanol. A 2^3^ factorial design was used to optimize the SLNs, which exhibited a PS of 320 nm, a PDI of 0.296, and an EE of 49.7%. The SLNs effectively delivered the drug to brain by intranasal administration in rabbits, as evidenced by gamma scintigraphic imaging [43]. Table 2 summarizes the major features of studies that utilized the solvent injection method, including the lipids used, drugs incorporated, and primary outcomes. 

## 4. Effect of Process Parameters on SLNs and NLCs Produced by Solvent Injection Method

First, we briefly introduce PS, PDI, EE, and DL of SLNs and NLCs prior to discussing the effects of preparation parameters. PS is a critical parameter for the process control and quality assurance of SLNs and NLCs preparations. The physical stabilities of SLNs and NLCs dispersions depend on PS since total surface area increases as PS decreases. Photon correlation spectroscopy (PCS) (or dynamic light scattering) and laser diffraction are the most powerful and widely used methods for measuring PS [55]. PCS measures PSs based on fluctuations in scattered light intensity, which are caused by particle movements. PS analysis using PCS is relatively sensitive and accurate, but its size range extends from a few nanometers to 3 µm [170]. On the other hand, laser diffraction can measure the PSs of nano-sized to lower millimeter-sized particles. This method is based on the relation between the diffraction angle and particle radius [170,171]. These two methods do not directly measure PS, as a calculation is required to determine them from light scattering effects [55]. If samples contain many size ranges, size measurements by PCS and laser diffraction may meet certain difficulties. In this case, light-weight microscopy may be an alternative option [37]. PDI is also an essential factor for evaluating SLNs and NLCs dispersions. A lower PDI value signifies a more monodispersed state [55]. Generally, a PDI value of <0.5 indicates a monodispersed, homogenous SLNs and NLCs dispersion, whereas a PDI value of >0.5 suggests non-homogeneity and polydispersity [37,172]. Most of the studies accept a PDI of <0.3 as an optimum value to indicate a good size distribution [172,173]. PDI can be measured by PCS [174,175]. EE and DL are parameters that reflect drug entrapment into the matrices of SLNs and NLCs. Increasing EE and DL is critical for SLNs and NLCs fabrication. EE is defined as the ratio between total drug incorporated into SLNs and NLCs and the amount of initial drug:

EE (%) = Total entrapped drug amount/Total drug amount × 100(1)

DL is the percentage of drug loaded in SLNs and NLCs [141,176] as defined by:

DL (%) = Amount of drug loaded/Amount of drug loaded and lipid added × 100 (2)

Several methods have been used to determine total amounts of entrapped drugs. Gel filtration chromatography (e.g., using Sephadex G-50 mini column) can be used to remove unentrapped drugs from SLNs or NLCs. Drugs are extracted from SLNs or NLCs dispersions and drug concentrations are analyzed by UV-Vis spectroscopy or high-performance liquid chromatography (HPLC) [148,156]. Dialysis membranes (e.g., 12–14 kDa) have also been employed to remove unentrapped drugs from SLNs or NLCs [168]. Ultracentrifugation is another method widely used. For example, SLNs or NLCs suspensions were ultracentrifuged twice at 60,000 × *g* for 1 h [147,152] or 35,000 × *g* for 40 min [177] at 4 °C to remove unentrapped drugs. In addition, a filter membrane (MWCO 10–20 kDa) can be used to separate SLNs and NLCs from unentrapped drugs. A sample is placed into a filter and put in a recovery chamber followed by ultracentrifugation (e.g., 15,000 rpm for 1 h). The free drug is then collected in the recovery chamber and analyzed [76,145].

Parameters affecting the production and properties of SLNs and NLCs can be classified as preparation process parameters and formulation parameters. Preparation process parameters include aqueous phase features (pH, temperature, and viscosity), organic phase features, the ratio of aqueous phase to organic phase, and dispersion characteristics (mechanical stirring, sonication). Formulation parameters include total lipid concentration, liquid lipid level, drug hydrophilicity, drug amount, emulsifier type, and emulsifier concentration. These parameters have different effects on the PSs, PDIs, EEs, and DLs of SLNs and NLCs.

### 4.1. Aqueous Phase

Several factors of aqueous phase, including pH, temperature, and viscosity, have different effects on SLNs and NLCs.

#### 4.1.1. pH

**Figure 2:**
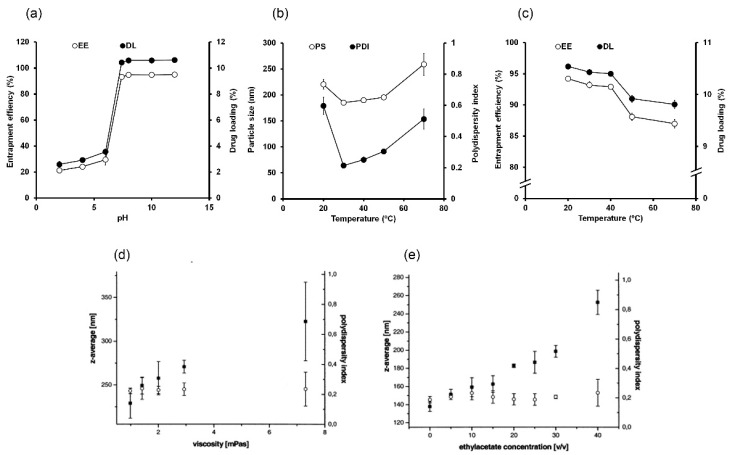
Effects of aqueous and organic phases on solid lipid nanoparticles (SLNs) and nanostructured lipid carriers (NLCs). (**a**) Effects of aqueous phase pH on entrapment efficiency (EE) and drug loading (DL) of ondansetron hydrochloride-loaded NLCs (data obtained from [76]). (**b**,**c**) Effects of aqueous phase temperature on particle size (PS), polydispersity index (PDI), EE, and DL of ondansetron hydrochloride-loaded NLCs (data obtained from [76]). (**d**,**e**) Effects of aqueous phase viscosity and ethylacetate concentration in the organic phase on PS (●) and PDI (○) of SLNs. Reprinted from [36] with permission from Elsevier.

The pH of an aqueous phase may critically influence SLNs and NLCs, particularly when drugs exhibit pH-dependent solubility. Several studies have reported the effects of aqueous phase pH on the EEs and DLs of SLNs and NLCs. U-86983, an anti-proliferative agent, is highly soluble under acidic conditions. During the preparation of U-86983-loaded SLNs, EE increased from 28.2 to 84.3% when the pH of the aqueous was increased from 6.5 to 8.4 [178]. Similarly, in another study, by adjusting the pH of the aqueous phase from 5.8 to 9.3, the EE of procaine hydrochloride increased from 11.0 to 58.2%, due to reduced drug solubility under alkaline conditions [179]. In the case of sodium diclofenac-loaded SLNs, EE reached ~100% when a pH 1.0 buffer solution was used because the drug is sparingly soluble under acidic conditions [176]. A previous study also clearly described the effects of aqueous phase pH on NLCs loaded with ondansetron hydrochloride [76]. Drug solubility was 31.7 mg/mL at pH 2.0, 41 µg/mL at pH 7.4, and 22–26 µg/mL at pH 8.0–12.0. Thus, the pH of the aqueous phase was altered to reduce drug leakage to the aqueous phase, which consequently enhanced EE and DL. When the pH was changed from 2.0 to 7.4, significant increases in EE and DL were observed (Figure 2a). In particular, the EE reached 93.2% at pH 7.4. No significant improvements in EE or DL were observed on further increasing aqueous phase pH since drug solubility was relatively low at pH values >7.4 [76]. Therefore, for drugs with pH-dependent solubility, the pH of the aqueous phase considerably affects the EE and DL values of SLNs and NLCs.

#### 4.1.2. Temperature

Aqueous phase temperature also critically influences the preparations of SLNs and NLCs, and temperature variation may change PSs, PDIs, EEs, and DLs. It was reported that the use of low temperature during the preparation of gonadorelin-loaded SLNs increase EE values [180]. In this case, the low temperature resulted in the rapid deposition of lipids in droplets. This deposition occurred at the interface between droplets and the aqueous medium, and prevented drug leakage to the outer aqueous phase. On the other hand, PS increased considerably when the temperature of the aqueous phase was reduced [172,180]. Similarly, in the case of ondansetron hydrochloride-loaded NLCs, at low temperatures (20 °C), PS and PDI were considerably increased, whereas EE and DL were improved (Figure 2b, c). Temperatures in mid-range (30–40 °C) resulted in low PSs, PDIs, and relatively high EEs and DLs. At high temperatures (e.g., 50–70 °C), PSs and PDIs increased since lipids were in the semisolid or molten state, and thus easily coalesced to create larger droplets [76], Whereas EEs and DLs of NLCs slightly decreased, as higher temperatures increased drug solubility and drug leakage to the aqueous phase [180]. In addition, at high temperatures, SLNs and NLCs might change to emulsion form and favor drug leakage [76,172]. Thus, optimum temperatures of the aqueous phase usually lie in the mid-range, though this depends on the properties of lipid mixtures. In the optimum temperature range, droplets containing drugs and lipids have enough time to break into smaller droplets prior to lipid precipitation. Furthermore, the temperature should not be too high to cause lipids to become molten or semisolid as this would probably facilitate drug leakage. 

#### 4.1.3. Viscosity

Usually, the aqueous phase used in the solvent injection method is water (or a buffer solution) containing an emulsifier at low concentrations (up to 2%) [42,44]. Thus, the viscosity of the aqueous phase is relatively low, which facilitates the diffusion of solutes from the organic phase. It was found in a previous study that increasing the viscosity of the aqueous phase (by adding glycerol) could increase PS by reducing diffusion rates (Figure 2d) [36].

### 4.2. Organic Phase

The organic phase consists of a drug and lipid mixtures dissolved in a water-miscible solvent or solvent mixture. Here, we summarize the influences of solvent type on SLNs and NLCs. For the solvent injection method, diffusion of organic solvent is a critical factor, and thus, appropriate solvent selection is important. Several solvents are frequently used, including ethanol, acetone, and isopropanol. In some cases, the solvents are mixtures of acetone and ethanol [38,161] or methanol and ethanol [39]. Some studies screened solvents for the preparation of SLNs and NLCs. For example, one group used ethanol, acetone, and isopropanol to prepare SLNs using Softosan^®^ 100 (a triglyceride mixture of fatty acids with chain lengths from C_10_ to C_18_) as solid lipid. Isopropanol was found to be the best in terms of PS, PDI, reproducibility, and reliability [36]. However, for SLNs prepared using glycerol monostearate, PSs increased in the order ethanol < isopropanol < acetone [172]. Thus, the effects of solvent are dependent on drug and lipid type. In addition, it should be noted that the miscibility of solvent and aqueous phases is a prerequisite of the solvent injection method. When ethylacetate was blended with isopropanol to prepare SLNs, PSs rose gradually with ethylacetate concentration (Figure 2e). In this case, since ethylacetate was not miscible with water, it decreased the diffusion coefficient of the solvent mixture and resulted in larger particles [36]. Thus, solvent choice is important in terms of controlling the PSs of SLNs and NLCs, and PSs can be adjusted by using different solvent or varying solvent blends. 

### 4.3. Ratio of Aqueous Phase to Organic Phase

**Figure 3:**
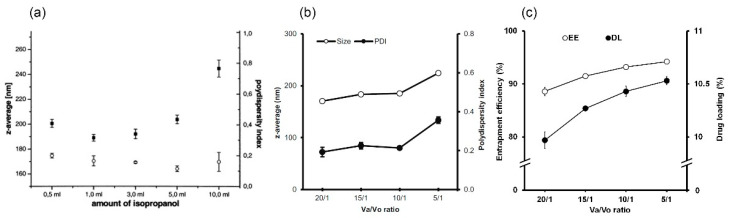
Effects of the ratio of aqueous phase to organic phase (Va/Vo ratio) on SLNs and NLCs. (**a**) Effects of Va/Vo ratio on PSs (●) and PDIs (○) of SLNs. The aqueous volume was 60 mL, whereas the volume of isopropanol was varied from 0.5 to 10 mL. Reprinted from [36] with permission from Elsevier. (**b**) Effects of Va/Vo ratio on PSs and PDIs of ondansetron hydrochloride-loaded NLCs. Reprinted from [76] with permission from Elsevier, Copyright (2019). (**c**) Effects of Va/Vo ratio on EEs and DLs of ondansetron hydrochloride-loaded NLCs, data obtained from [76].

Overall, the relative ratio of aqueous phase to organic phase (Va/Vo) does not considerably affect PSs or PDIs unless it exceeds a critical value. For example, in the case of SLNs prepared with Sotisan^®^ 100, the Va/Vo ratio could be changed from 60/1 to 6/1 without considerable effects on PS and PDI (Figure 3a). However, a decrease in Va/Vo ratio from 6/1 to 3/1 resulted in a PS increase from ~200 nm to ~250 nm and a slight rise in PDI [36]. In a recent study, it was found that when the Va/Vo ratio was decreased from 10/1 to 5/1, PS was not significantly changed (279 to 278 nm), and PDI slightly decreased (0.39 to 0.36). However, on further decreasing Va/Vo to 2.5/1, PS and PDI significantly increased (PS to 370 nm and PDI to 0.81) [169]. Another study also highlighted the effects of Va/Vo ratio on PS, PDI, EE, and DL (Figure 3b,c). As Va/Vo ratio decreased from 20/1 to 5/1, PS, PDI, EE, and DL increased [76]. A reduction in aqueous phase volume reduces the diffusion of organic solvent out of droplets and promotes the formation of larger particles with varying sizes [36]. However, it also reduces drug migration to the aqueous phase and increases EE and DL [76]. In practical experiments, a suitable range of Va/Vo ratios can be determined for individual systems. It is worth noting that at high Va/Vo ratios, the produced SLNs and NLCs have small PSs and narrow size distributions. However, in these cases, EE and DL are reduced and greater amounts of water and surfactant are required. Furthermore, high surfactant levels are not preferred when applying the SLNs and NLCs system to certain administration routes such as parenteral administration. Furthermore, greater amount of water makes SLNs and NLCs dispersions very diluted [3]. Therefore, the selection of an appropriate Va/Vo ratio is probably best based on PS, PDI, EE, and DL values. As long as Va/Vo ratios are higher than critical values, they have minor effects on PS and PDI [36], and thus, a lower Va/Vo ratio should be considered as it markedly improves EE and DL values.

### 4.4. Dispersion Energy

Sonication is frequently used to supply dispersion energy to produce SLNs and NLCs. It has been reported that when the sonication time is varied, observed effects are slight. For example, as sonication time was increased from 2 to 4 and 6 min, PS decreased from 306 to 206 and 198 nm and PDI increased significantly from 0.18 to 0.21 and 0.45, respectively [160]. In another study, sonication for 2–4 min slightly reduced PS and PDI as compared with the case of no sonication. Upon increasing the sonication time from 4 to 6 min, PS remained unchanged, whereas PDI slightly increased as a result of irregular size reduction [76]. In these studies, the optimal sonication time was 4 min [76,160]. The effects of sonication time on EE and DL reported in two studies differed. One study showed that a longer sonication time reduced EE (from 92.8% at 2 min to 90.9% at 4 min and 80.4% at 6 min) [160], whereas the other reported sonication time had negligible effects on EE and DL [76]. These differences may have been due to the process temperatures, which were 70 and 30**°**C, respectively. At high temperature, sonication energy may facilitate the expulsion of drug molecules from emulsion droplets [160], whereas at low temperatures, the lipid quickly precipitates and prolonged sonication may not cause drug leakage [76]. Overall, the use of short sonication times (2–4 min) is favored for reducing PS and PDI slightly. Longer sonication times should be carefully evaluated because they may have undesirable effects, such as increased PS and PDI or decreased EE and DL.

## 5. Effect of Formulation Parameters on SLNs and NLCs Produced by Solvent Injection Method

### 5.1. Total Lipid Concentration

To investigate the effects of total lipid concentration in the organic phase on SLNs and NLCs, one can vary total lipid concentration while holding other variables constant. In general, a high lipid concentration increases PS since it increases organic phase viscosity and reduces the diffusion rate of lipid molecules to the outer phase. For example, in a study of Schubert et al., as lipid concentration was increased from 10 to 40 mg/mL, PS increased considerably from ~140 to ~210 nm [36]. Joshi et al. reported an increase in PS of SLNs from 320 to 360 nm when the lipid concentration was increased from 10 to 15 mg/mL [43]. Similarly, increasing concentration of glycerol monostearate from 50 to 100 mg/mL resulted in a considerable increase in PS (from 272 to 315 nm) [166], whereas changing stearic acid concentration from 40 to 50 mg/mL caused an increase in PS (282 to 305 nm) [169]. PDI is also affected by total lipid concentration. When total lipid concentration was increased from 20 to 60 mg/mL, PDI was not appreciably affected, but a further increase to 80 mg/mL significantly increased PDI [76]. Similarly, increasing stearic acid concentration from 40 to 50 mg/mL caused increase in PDI (0.32 to 0.69) [169]. In these cases, increases in lipid concentration and organic phase viscosity reduced agitation efficacy and increased particle agglomeration [172], which broadened the size distribution [85,159]. 

When the drug amount is kept constant, increasing the total lipid amount creates more space for drug accommodation, and thus, increases EE. A study on SLNs preparation showed that EE rose slightly from 49.7 to 56.6% as the lipid concentration was increased from 10 to 15 mg/mL [43]. Similarly, in the case of simvastatin-loaded SLNs, varying the concentration of lipid from 50 to 100 mg/mL increased EE from 69.7 to 80.9% [166]. When the drug/total lipid ratio is constant, initial drug input and total lipid amount are both increased. One might infer that since the compositions of the SLNs and NLCs are unchanged, EE and DL would not change appreciably. However, as the lipid concentration is increased, organic phase viscosity increases, and this reduces the diffusion rate of solute molecules (i.e., reduces drug leakage to aqueous phase). In addition, the time required for the formation of lipid nanoparticles may reduce, which decreases drug leakage to the aqueous phase during lipid precipitation and increases EE and DL. It was found that EE and DL increased (81.1 to 94.5% and 9.20 to 10.56%, respectively) when the lipid concentration was increased (from 20 to 80 mg/mL) [76]. Therefore, total lipid concentration plays a critical role during the preparation of SLNs and NLCs. High lipid concentrations increase EE and DL, but are associated with the formation of larger particles with high PDIs. Furthermore, increasing lipid concentration increases the risk of lipid precipitation in the organic phase during preparation. On the other hand, the use of a low lipid concentration resulted in small and narrow-distributed SLNs and NLCs, but it is associated with reductions in EE and DL [76]. Therefore, it is important to use an appropriate concentration of lipid in the organic phase. As long as PS and PDI remain within accepted ranges, this concentration can be maximized. Table 3 summarizes the effects of total lipid concentration on SLNs and NLCs as reported in several studies.

### 5.2. Liquid Lipid Level

As previously reported, when the liquid lipid amount (soybean lecithin + medium-chain triglycerides (MCT)) was increased from 0 to 40% (on total lipid added), PS and PDI significantly decreased. In detail, for 0% liquid lipid, PS was 479 nm and PDI was 0.441. These numbers changed to 213 nm (PS) and 0.234 (PDI) for 20% liquid lipid and to 185 nm (PS) and 0.214 (PDI) for 40% liquid lipid [76]. In another study, the PS of SLNs decreased considerably from 426 to 311 nm and PDI reduced from 0.38 to 0.24 when soya lecithin concentration was increased from 20 to 50% [160]. Similar results have been reported for oleic acid [42,181], MCT [4], and phosphatidylcholine [169]. The smaller PS and PDI of NLCs produced at higher liquid lipid levels are attributed to reductions in lipid blend viscosity [4,42,182]. However, there are upper limits for liquid lipid level that depend on the natures of solid and liquid lipids. In the case of tripalmitin (solid lipid) and soybean lecithin plus MCT (liquid lipid), increasing liquid lipid to 50% caused PS and PDI to increase significantly (185 to 213 nm and 0.214 to 0.521, respectively) [76]. When the liquid lipid level was exceeded, it leaked into the aqueous phase, resulting in the formation of mixed micelles, liposomes, and other structures [160,176]. Similarly, as reported by Jain et al., when the soya lecithin percentage in a lipid matrix with tristearin was increased from 50 to 56%, PS increased slightly (311 to 360 nm) and PDI increased remarkably (0.24 to 0.44) [160]. In another study, increasing the level of distearoyl-phosphatidylethanolamine from 50 to 67% also led to increases in PS (195 to 233 nm) and PDI (0.265 to 0.404) [155]. Overall, liquid lipid level in an appropriate range is one of the parameters largely responsible for decreases in PS and PDI. 

Several studies have investigated the effects of liquid lipid levels on EEs and DLs of NLCs. It was found that upon increasing oleic acid levels from 15 to 30 mg, the EE of NLCs increased significantly from 83.9 to 93.3% [42]. Investigations by another group showed a slight increase in EE when MCT was added to the lipid matrices of NLCs [183]. A previous report pointed out that the addition of MCT (a liquid lipid) to Compritol^®^ 888 ATO (a solid lipid) at 50% showed higher EE than SLNs (88% versus 80%) [4]. It was also found that both EE and DL increased on increasing liquid lipid level [76]. The presence of liquid lipids in the matrices of NLCs offer more space for drug molecules, due to the formation of less-ordered structures in NLCs [4,184]. In summary, the incorporation of liquid lipids at appropriate levels is beneficial to NLCs with respect to PS, PDI, EE, and DL. The effects of liquid lipids on NLCs are briefly described in Table 4.

### 5.3. Drug Hydrophilicity and Drug Amount

The solubility of a drug in water is a critical consideration for SLNs and NLCs preparation using the solvent injection method. During preparation, drugs may leak to the aqueous phase, depending on its aqueous solubility, which results in low EE and DL values for some hydrophilic drugs [20,43]. When using the same lipid matrix and preparation method, hydrophilic drugs have significantly lower EEs than hydrophobic drugs, as exemplified by 5-fluorouracil (EE 13.2–15.6%) and cinnarizine (EE 98.9–100%) [141]. This is the reason why SLNs and NLCs are more suitable for hydrophobic drugs. In some cases, as discussed above, drugs with pH-dependent solubility may achieve high EEs when the pH of the aqueous phase is adjusted [76]. 

The initial amount of drug used critically influences SLNs and NLCs. It was reported that increasing the amount of initial drug added (ondansetron hydrochloride) from 6.3% to 12.5% resulted in negligible increases in PSs (172–185 nm) and PDIs (0.191–0.214). However, when this was further increased to 16.7%, PS increased to 254 nm and PDI to 0.509. In this case, the lipid matrix may have been saturated with the drug, leading to particle aggregation [76]. A similar result was reported with miconazole nitrate-loaded SLNs. In the drug concentration range 2.5–7.5%, PS and PDI remained almost constant, but when the drug concentration was increased to 10.0%, remarkable increases in PS and PDI were observed (from 329 to 353 nm and from 0.26 to 0.41, respectively) [160]. In another case when SLNs were loaded with paclitaxel, increasing the drug level from 10 to 20% resulted in a significant increase in PS (from 195 to 318 nm) [155]. Increasing drug concentration in SLNs and NLCs can also change the pH of the aqueous medium, particularly when drugs possess pH-dependent groups, and these pH changes may result in drug precipitation and increase PS and PDI. Thus, when these drugs are used, the aqueous phase is usually buffered to maintain pH. In addition, the amount of drug present is usually far less than the amount of aqueous phase. In such cases, aqueous phase pH would not be significantly altered by increasing drug concentration [76,176].

The effects of drug amount on EE and DL are more prominent. In the case of NLCs loaded with ondansetron hydrochloride, as the initial drug amount was increased from 6.3% to 16.7%, more drug molecules were entrapped in the lipid matrix, which increased DL (5.1 to 12.2%). EE also increased (from 86.7 to 93.2%) when the initial amount of drug added was increased from 6.3% to 12.5%. However, a further increase in the initial amount of drug added (to 16.7%) reduced EE to 83.3%, which may have been due to an increase in the amount of drug lost to the aqueous phase [76]. Similarly, increases in the amount of paclitaxel (0.05 to 0.25 mmol) enhanced EE (72.2 to 89.0%) and DL (16.3 to 25.0%). However, when the amount of drug added was increased to 0.5 mmol, EE and DL reduced to 66.5% and 23.6%, respectively, since the maximum loading capacity of the system was reached and the extra paclitaxel precipitated [165]. This group reported similar findings in a later study, that is, decreases in EE (75.4 to 53.0%) and DL (31.5 to 18.1%) when drug content was increased from 0.25 to 0.5 mmol [147]. Another study on paclitaxel-loaded NLCs showed a similar pattern. EE increased (74.1 to 84.5%) upon increasing initial drug amount (5 to 10%), but gradually decreased at high initial drug amounts (15–20%) [155]. In the case of miconazole nitrate-loaded SLNs, increasing the drug amount from 2.5 to 7.5% increased EE from 87.6 to 92.7%, but further increase to 10% reduced EE to 84.9% [160]. Increases in drug amounts have been suggested to produce more porous structures (with large channels and hollows) that allow drug molecules to escape to the aqueous phase [185]. In addition, increased drug amounts inside nanoparticles would increase osmotic pressure difference between the aqueous and organic phases, which cause droplet damage, and thus increase drug leakage [186]. Therefore, for each system, there is a critical drug amount. Above this value, EE starts to decrease on increasing initial drug amounts [176]. It should be noted that when the initial drug amount is higher than the critical value, although EE decreases, absolute amounts of drug entrapped inside SLNs and NLCs increase, and this leads to a continuous increase in DL [76,176]. Thus, initial drug amounts critically affect the properties of SLNs and NLCs, particularly EE and DL. There is a range in which increasing initial drug amount improves EE and DL and does not significantly change PS or PDI. One may select highest initial drug amounts in this range to incorporate drugs into SLNs and NLCs. Table 5 presents the major findings of some studies that have addressed this topic.

### 5.4. Emulsifier

Several studies have reported the effects of emulsifiers on SLNs and NLCs. For example, when no emulsifier was used, ondansetron hydrochloride-loaded NLCs had a PS of 239 nm and a high PDI value (0.542). However, at a low concentration of polysorbate 80 (0.1%), PS decreased to 185 nm and PDI to 0.214 [76]. Similarly, the use of polysorbate 80 in the aqueous phase at 0.1% resulted in a PS reduction from ~200 to ~160 nm [36]. Thus, it appears that emulsifiers play an essential role in the formation of SLNs and NLCs, as they reduce interfacial tension between the organic phase and the aqueous phase, which results in the initial formation of smaller solvent droplets at injection sites [36,160]. In addition, the emulsifier contributes to droplet stability and prevents droplet coalescing. When no emulsifier is used, initial droplets might be broken into smaller particles with varying sizes, which would increase the size distribution [43].

Changes in PS and PDI have been reported when the concentrations of emulsifiers are changed. As lecithin concentration was increased from 0.5 to 1.0%, both PS and PDI decreased (352 to 320 nm and 0.446 to 0.296, respectively) [43]. PSs also significantly decreased (from 292 to 220 nm) upon increasing poloxamer 407 concentration from 0.8 to 2% [166]. Jain et al. showed that an increase in polysorbate 80 concentration from 0.1 to 2.0% reduced PSs from 329 to 255 nm. PDIs first decreased from 0.26 to 0.18 as the emulsifier concentration was changed from 0.1 to 0.5%, and then increased to 0.46 and 0.56 at emulsifier concentration of 1.0 and 2.0%, respectively [160]. 

**Figure 4:**
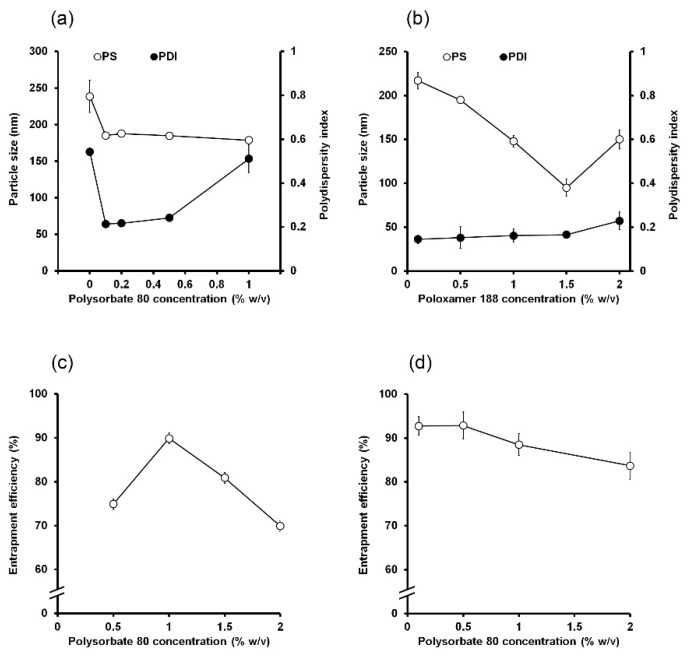
Effects of emulsifier on SLNs and NLCs. (**a**) Effects of polysorbate 80 concentration on PSs and PDIs of NLCs, data obtained from [76]. (**b**) Effects of poloxamer 188 concentration on PSs and PDIs of SLNs, data obtained from [165]. (**c**,**d**) Effects of polysorbate 80 concentration on EEs of SLNs, data obtained from [161] (for (**c**)) and [160] (for (**d**)).

In another study, increasing polysorbate 80 concentration from 0.1% to 0.5% did not significantly affect the PSs or PDIs of NLCs (Figure 4a). However, a further increase to 1% resulted in a significant PDI increase (0.511) [76]. In the case of adapalene-loaded SLNs, an increase in polysorbate 80 concentration from 0.5 to 1.5% decreased PS (219–142 nm, but PS and PDI values increased as the emulsifier concentration was further increased to 2.0% [161]. Similarly, as shown in Figure 4b, when poloxamer 188 concentration was varied from 0.1 to 1.5%, the PS of SLNs decreased from 217 to 95 nm, whereas PDI increased slightly (0.145–0.166). On the other hand, the use of poloxamer 188 at a higher concentration (2%) significantly increased PS (to 150 nm) and PDI (to 0.229) [165]. In another study, similar results were found. On varying poloxamer 188 concentration from 0.1 to 1.5%, PS reduced (244–70 nm) and PDI increased slightly (0.141–0.165). At an emulsifier concentration of 2%, PS and PDI significantly increased (to 131 nm and 0.21, respectively) [147]. A recent study by Eleraky et al. showed that increasing emulsifier concentration from 1 to 5% caused PS and PDI to increase significantly [153]. However, in this study, the emulsifier range did not include low concentrations (0.1–0.5%), whereas the optimum emulsifier concentration may have been around 0.5–1.5%. Many authors have pointed out that increases in emulsifier level initially reduce PS and PDI, but after a critical value is reached, emulsifier additions increase PS and PDI. There are two primary explanations for this behavior. One is that lipid molecule diffusion rates are reduced as the viscosity of the outer phase increases with increased emulsifier concentration [165]. The other one relates to the irregular size reduction of SLNs and NLCs, which increases PDI [160,161]. 

The effects of emulsifier concentration on EE and DL are different in several studies. One study reported that emulsifier concentration had only minor influences on EE and DL [76], whereas others found that high emulsifier levels reduced EE and DL. As polysorbate 80 concentration was increased from 1.0 to 1.5 and 2.0%, EE decreased from 89.9 to 80.9 and 69.9%, respectively (Figure 4c) [161]. Similarly, as shown in Figure 4d, increasing polysorbate 80 levels from 0.5 to 1.0 and 2.0% reduced EE from 92.8 to 88.5 and 83.7%, respectively [160]. In another study, upon an increase in poloxamer 407 concentration from 0.8 to 1.2%, EE was almost unchanged (83.9–82.9%) or decreased slightly (93.3–86.5%) [42]. However, Shah et al. showed that EE significantly decreased (from 78.9 to 65.8%) upon increasing poloxamer concentration from 0.8 to 2% [166]. Although there are differences in the literature, emulsifiers are considered crucial for the preparation of SLNs and NLCs using the solvent injection method. The use of emulsifiers at high concentrations does not favor SLNs or NLCs formation due to increases in PS and PDI, and in some cases, increases in EE and DL. Thus, emulsifiers should be used in their appropriate ranges, usually 0.1–0.5%, and if possible, at low concentrations. 

## 6. Authors’ Outlook and Conclusion

SLNs and NLCs have attracted the interest of scientists for more than 20 years because of their important drug delivery applications. Many methods have been developed and investigated to prepare SLNs and NLCs [1]. High-shear homogenization and high-pressure homogenization are two solvent-free methods used for SLNs and NLCs preparation. The former is one of the methods initially used and the latter is a reliable and effective technique for the large-scale preparation of SLNs and NLCs. However, they require sophisticated instruments and expose drugs to high temperatures for extended periods [75]. Other methods, such as emulsion/solvent evaporation, solvent diffusion, emulsification sonification, phase inversion, and microemulsion-based methods, have some disadvantages. For example, they require organic solvents and high surfactant levels, and involve complicated and long processes, or the dilution of SLNs and NLCs dispersions [187,188]. The solvent injection method provides an alternative to these frequently used methods. It requires only simple equipment that is normally available in laboratories, and is straightforward and rapid [37]. When drugs are sensitive to high temperatures, the solvent injection method provides an appropriate option. Furthermore, it is applicable to both hydrophobic [150,189] and hydrophilic drugs [38,76]. The main drawback of the method is that it requires organic solvents [1], and is not easily scaled up. 

The solvent injection method has not been widely utilized and investigated due to the feasibilities of other methods, such as high-shear homogenization and high-pressure homogenization. Nevertheless, studies have evaluated the use of this method for the production of SLNs and NLCs. Many preparation process and formulation variables determine the properties of SLNs and NLCs. PS, PDI, EE, and DL can be adjusted by varying these variables. When the solvent injection method is used to prepare SLNs and NLCs, the following issues require consideration.

Some solvents such as ethanol, acetone, isopropanol, and their mixtures are commonly used and should be screened to determine which is the most appropriate solvent for a given lipid-drug system [36,153].

When a drug exhibits pH-dependent solubility, the pH of the aqueous phase should be adjusted to improve EE and DL [76].

Some variables, including Va/Vo ratio, sonication time, total lipid amount, liquid lipid level, drug amount, and emulsifier concentration, have different effects on PS, PDI, EE, and DL, and should be optimized for each drug and lipid matrix [147,165]. Mid ranges of these variables normally result in SLNs and NLCs with desirable properties.

The various preparation methods mentioned have advantages and disadvantages. Many factors should be considered when choosing an appropriate method to prepare SLNs and NLCs. Of the methods that are frequently used and have been well investigated, solvent injection provides the ability to control the PS, PDI, EE, and DL values of the products and can be used for both hydrophilic and hydrophobic drugs [76,150]. This simple, rapid method can be easily used in the laboratory to prepare SLNs and NLCs for preclinical evaluations. The use of organic solvents, a major drawback of this method, can be addressed by using solvent removal methods, such as lyophilization [190] or spray-drying [191]. In many cases, the solvent is ethanol and can be used as a component of topical formulations [160]. In summary, this review presents most of the critical issues concerning the preparation of SLNs and NLCs using the solvent injection method. Studies on SLNs and NLCs are certain to be performed due to their potential applications for drug delivery. In addition to chemical compounds, SLNs and NLCs can deliver peptides and proteins with a high degree of efficiency and low toxicity [102,103,104]. They are also potential platforms for non-viral gene delivery [192,193]. A recent study prepared siFVII-loaded NLCs for intravenous injection in mice, and achieved >80% knock-down of plasma coagulation factor VII in liver [144]. The solvent injection method requires further investigation as a means of preparing SLNs and NLCs at commercial levels. The micro-channel with cross-shaped junction [142] and co-flowing micro-channel [143] systems are of particular interest in this respect. In addition, the large-scale use of baffle devices that allow on-device post-treatment [144] also requires investigation.

## Figures and Tables

**Table 1:** Mechanisms, advantages, and disadvantages of different SLNs and NLCs preparation methods.

Method	Mechanism	Advantage	Disadvantage
Hot high-pressure homogenization	High shear stress and cavitational forces	Speed, straightforward, avoidance of organic solvents, scalability	Drug degradation under high temperature, drug loss into the aqueous phase
Cold high-pressure homogenization	High shear stress and cavitational forces	Prevention of drug degradation, applicability to hydrophilic drugs	Large particles, broad size distributions
High-speed stirring and ultra-sonication	High shear between two solid adjacent area Formation, growth, and implosive collapse of bubbles in a liquid	straightforward, avoidance of organic solvents, low cost, scalability	Exposure of drugs to high temperatures, metal contamination from sonicator probes, high surfactant concentrations, low lipid concentrations
Microemulsion	Spontaneous interfacial tension reduction under dilution	Simplicity, reproducibility, scalability, avoidance of organic solvents	Large amount of water to dilute microemulsions, high concentration of surfactants
Solvent emulsification-diffusion	Diffusion of solvent from lipid phase to aqueous phase leading to lipid precipitation	Simplicity, avoidance of heat, small PS, narrow size distribution Scalability, applicability to both hydrophilic and hydrophobic drugs	Residual solvent, additional solvent removal procedures
Solvent emulsification-evaporation method	Evaporation of solvent in lipid phase leading to lipid precipitation	Simplicity, avoidance of heat, small PS, narrow size distribution	Residual solvent, additional solvent removal procedure Dilute suspensions, requirement of evaporation or ultra-filtration
Double emulsion	Lipid crystallization due to solvent evaporation or low temperature	Applicability to hydrophilic drugs	Low EE and DL Large PS
Phase inversion temperature (PIT)	Spontaneous inversion between oil/water and water/oil emulsions with temperature change	Low energy, avoidance of organic solvents, narrow size distribution, good stability	Instability of emulsion
Membrane contactor	Formation of small droplets after pressing lipid phase through membrane pores	Scalability, control of size	Clogging of membrane
Supercritical fluid-based methods	Quick evaporation or diffusion of solvent with the help of supercritical fluid, resulting in lipid precipitation	Uniform particle size distribution, high solvent extraction efficiency	Use of organic solvent, high expense
Coacervation	Precipitation of alkaline salts of fatty acids when decreasing pH	Simplicity, no sophisticated instrument, avoidance of organic solvents	Applicability only to lipids in alkaline salt form and non pH-sensitive drugs
Solvent injection	Diffusion of solvent from lipid phase to aqueous phase leading to lipid precipitation	Simplicity, straightforward, fast production process, no sophisticated instrument	Residual solvent, additional solvent removal procedure

PS: particle size, EE: entrapment efficiency, and DL: drug loading.

**Table 2:** Overview of lipids, emulsifiers, drugs, and solvents used to prepare SLNs and NLCs by solvent injection method and their significant outcomes.

Lipid(s)/Emulsifier(s)	Drug or Active Ingredient	Solvent	Outcomes	Year, Reference
Lipoid^®^S 100/sucrose fatty acid ester	Paclitaxel	Acetone	PS: 188 nm, PDI: 0.396, and EE: 92.2%SLNs showed sustained-release for 14 days (in vitro)	2006, [40]
Palmitic or stearic acid/phosphatidylcholine	Idebenone	Ethanol	PS: 170–183 nm, PDI: 0.113–0.134, and EE: 83–86% SLNs showed sustained-release for 7 days (in vitro) SLNs increased drug protecting activity against free radical-induced oxidative damage of astrocyte cells	2006, [41]
Stearic acid, soya lecithin/poloxamer 188	Paclitaxel	Diethyl ether	Drug amount and emulsifier concentration were varied to get the optimized SLNs (PS: 113 nm, PDI: 0.156, and EE: 89.0%) SLNs had lower toxicity on HepG2 cell than free drug SLNs was stable for 90 days	2009, [165]
Tristearin, soya lecithin/polysorbate 80	Miconazole nitrate	Ethanol	Parameters were varied to obtain optimized NLCs (PS: 206 nm, PDI: 0.21, and EE: 90.9%) NLCs-based hydrogels increased skin retention 10-fold as compared with miconazole suspension and miconazole hydrogel	2010, [160]
Tristearin/polysorbate 80	Hepatitis B surface antigen (HBsAg)	Acetone	HBsAg was loaded onto SLNs surface Mannosylated SLNs (PS: 96.6 nm, PDI: 0.05, and EE: 64.5%) SLNs showed better cellular uptake, lesser toxicity, and greater immune response Subcutaneous administration of SLNs was potential for vaccine delivery against hepatitis B	2010, [159]
Monostearin/poloxamer 407	Simvastatin	Isopropanol	A 2^3^ factorial design was performed to optimize SLNs (PS: 259 nm, EE: 75.8%) SLNs showed sustained-release for 55 h (in vitro)	2010, [166]
Tristearin, soya lecithin/polysorbate 80	Doxorubicin hydrochloride	Acetone + ethanol	Mannosylated SLNs (PS: 360 nm, PDI: 0.135, and EE: 70.3%) Mannosylated SLNs showed sustained-release in mice. They delivered a higher dug concentration to the tumor mass.	2010, [38]
Glycerol monostearate, oleic acid/poloxamer 407	Simvastatin	Isopropanol	A 2^3^ factorial design was performed to optimize NLCs (PS: 212 nm, PDI: 0.344, and EE: 84%) NLCs showed a higher bioavailability than drug suspension and drug-loaded SLNs	2011, [42]
Monostearin, soya lecithin/poloxamer 188	Puerarin	Methanol + ethanol	SLNs enhanced oral bioavailability of the drug 3 times Tissue concentration of the drug increased, particularly the hearts and brain after oral administration of SLNs	2011, [39]
Stearylamine, soya lecithin, α-tocopherol/poloxamer 188	Paclitaxel	Diethyl ether	Drug amount and emulsifier concentration were varied to obtain optimized SLNs (PS: 96 nm, PDI: 0.162, EE: 75.4%, and DL: 31.5%) SLNs increased oral bioavailability of the drug 10 times in mice	2011, [147]
Monostearin/Lecithin + poloxamer 188	Ondansetron hydrochloride	Ethanol	A 2^3^ factorial design was performed to get the optimized SLNs (PS: 320 nm, PDI: 0.296, and EE: 49.7%) The SLNs effectively delivered the drug to the brain by intranasal administration on rabbits	2012, [43]
Stearic acid/polysorbate 80	Cytarabine	Isopropanol	Drug and lipid was conjugated prior to SLNs preparation - PS: 137 nm, PDI: 0.151, and EE: 58.4% SLNs showed drug sustained-release (3 days in vitro) and increased toxicity on leukemic EL-4 cells as compared with drug solution	2012, [167]
Glyceryl behenate (Compritol^®^ 888 ATO)/poloxamer 407	Terbinafine hydrochloride	Isopropanol	A 3^3^ factorial design was performed to get the optimized SLNs (PS: 274 nm, PDI: 0.32, and EE: 74.6%) SLN-based gel was more effective than a commercial product when applying in rats (pharmacodynamics studies) - SLNs were stable for 90 days	2013, [44]
Monostearin/polysorbate 80 + poloxamer 188	Thymoquinon	Ethanol	Box-Behnken design was used to optimize the SLNs (PS: 166 nm, EE: 71.6%) Oral bioavailability increased 5-fold in rats as compared with drug suspension	2013, [145]
Dynasan 114, soya phosphatidylcholine/poloxamer 407	Adefovir dipivoxil	Isopropanol	Different process and formulation parameters were evaluated to optimize SLNs (PS: 267 nm, EE: 73.5%, and DL: 2%) SLNs increased oral bioavailability 2- and 1.5-fold as compared with micro-suspension and nanosuspension, respectively SLNs increased drug accumulation in liver, kidneys, intestine, and stomach.	2013, [149]
Cetyl alcohol/polysorbate 80	Andrographolide	Ethanol	PS: 154 nm, PDI: 0.172, EE: 91.4%, and DL: 18.6% SLNs increased oral bioavailability (3.41-fold) and antitumor activity as compared with the drug suspension	2014, [150]
Tristearin, soya lecithin/polysorbate 80	Adapalene	Acetone + ethanol	PS: 148 nm, PDI: 0.169, and EE: 89.9% SLNs-based gel showed sustained-release in vitro	2014, [161]
Tristearin, hydrogenated soya phosphatidylcholine/polysorbate 80	Aceclofenac	Ethanol	SLNs was conjugated with chondroitin sulfate (CS-SLNs) with PS: 154 nm, PDI: 0.403, and EE: 65.4% SLNs and CS-SLNs increased drug amount in the inflammatory knee joint (2- and 10-fold as compared with the drug solution, respectively) after IV administration in rats	2014, [156]
Monostearin/polysorbate 80	Halobetasol propionate	Isopropanol	A 3^2^ full factorial design was applied to optimize the SLNs (PS: 200 nm and EE: 93%) SLNs-based carbopol gels prolonged drug release up to 12 h on human cadaver skin, reduced systemic uptake, increased drug accumulation in skin, and were nonirritant to rabbit skin	2014, [162]
Monostearin or stearic acid/polysorbate 80 or poloxamer 188	Tamoxifen	Methanol	Lipid and emulsifier were varied to obtain optimized SLNs (PS: 130 nm, PDI, 0.231, and EE: 86.1%) SLNs increased oral bioavailability (1.6-fold) following oral administration in rats	2014, [151]
Monostearin, Tefose-63/polysorbate 80	Mometasone furoate	Ethanol	SLNs was optimized (PS: 124 nm, and EE: 55.6%) SLNs-based carbopol gel increased skin deposition 2.67- and 20-fold as compared with a marketed cream and drug-loaded gel SLNs-based gel increased skin permeability 15.2-fold as compared with a marketed cream	2014, [163]
Tristearin, distearoyl-phosphatidyl ethanolamine/polysorbate 80	Paclitaxel	Acetone + ethanol	Mannosylated SLNs (PS: 254 nm, PDI: 0.312) Mannosylated SLNs improved antiproliferative efficacy in lung cancer cells. They delivered a higher drug concentration to alveolar cells after IV injection in rats	2015, [155]
Tristearin, soya lecithin, stearylamine/polysorbate 80	Rifampicin	Ethanol	Drug-loaded SLNs was coupled with lactoferrin to enhance SLNS delivery to lung (PS: 271 nm, PDI: 0.124, and EE: 68.4%) In vivo biodistribution study: lactoferrin-coupled SLNs had 47.7% drug uptakes by the lungs (3.05 times higher than uncoupled SLNs) following IV injection in rats	2015, [157]
Tripalmitin/polysorbate 80	Sumatriptan	Ethanol	A 2^3^ randomized full factorial design was performed to optimize the SLNs (PS: 236 nm, and EE 91.3%) SLNs showed a 4.54-fold increase in brain/blood ratio of drug (2 h after oral administration in rats) SLNs improved anti-migraine potential in behavioral studies	2015, [152]
Tristearin, hydrogenated soya phosphatidylcholine/polysorbate 80	Nifedipine	Ethanol	SLNs was further coated with polysorbate 80 (PS: 121 nm, PDI: 0.261, and EE: 71.5%) Coated-SLNs increased bioavailability about 5- and 2-fold as compared with free drug and uncoated SLNs, respectively (IV administration in rats) Coated-SLNs increased drug accumulation in brain	2015, [158]
Compritol^®^ 888 ATO, Gelucire^®^ 50/13/polysorbate 80	Resveratrol	Ethanol	Box–Behnken design was applied to optimize SLNs (PS: 191 nm, PDI: 0.156, and EE: 73.7%) SLNs increased oral bioavailability nearly 5-fold in rats as compared with resveratrol suspension Pharmacodynamic data showed a decrease in the serum biomarker enzymes as compared with control and marketed formulation (against paracetamol-induced liver cirrhosis)	2016, [146]
Monostearin/poloxamer 188	Asiatic acid	Ethanol	A Box–Behnken design was used to optimize the formulations (PS: 237 nm, EE: 64.4%, and DL: 31.9%) SLN increased oral bioavailability 2.5-fold following oral administration in rats	2016, [148]
Vitamin B6-stearic acid conjugation/polysorbate 80	Doxorubicin	Ethanol	Vitamin B6 was conjugated with lipid to modify charge of SLNs (PS: 114 nm, PDI: 0.101, and DL: 7.1%) Vitamin B6-modified SLNs had an increased therapeutic efficacy and lower toxicity in tumor-bearing rats as compared with free drug Vitamin B6-modified SLNs prolonged drug circulation in blood and increased drug accumulation to tumor site in rats	2016, [154]
Sophorolipid/poloxamer 407 and 188	Rifampicin + dapsone	Ethanol	Five different polymers were used to stabilize SLNs, and poloxamer 407 and 188 were the best options - There was no in vivo study	2018, [168]
Tripalmitin, Phosal^®^ 53MCT/polysorbate 80	Ondansetron hydrochloride	Ethanol	Various parameters were investigated to get the optimized NLCs (PS: 185 nm, PDI: 0.214, EE: 93.2%, and DL: 10.43%) NLCs showed sustained-release in vitro and in vivo for up to 4 days following subcutaneous administration in rats	2019, [76]
YSK05, cholesterol, DMG-PEG2K	siRNA	Ethanol	NLCs were subjected to post-treatment using an integrated baffle device (PS: 33 nm and EE: 90%) The siFVII knocked down the plasma coagulation factor VII at mice liver tissue more than 80% (IV injection, mice)	2020, [144]
Compritol^®^ 888 ATO, oleic acid/poloxamer 407	Temazepam	Acetone + ethanol	A 4^2^ full factorial design was applied to optimize NLCs (PS: 307 nm, PDIL 0.09, and EE: 75.2%) NLCs increase oral bioavailability nearly 3-fold in rats as compared with temazepam suspension NLCs improved brain uptake of ^99m^Tc-temazepam	2020, [153]
Stearic acid, phosphatidylcholine	Alpha-tocopherol	Ethanol	Various parameters were evaluated to optimize SLNs (PS: 175 nm, EE: 90.9%, and DL: 59.4%)No in vivo study was conducted	2020, [169]

Particle sizes (PSs), polydispersity indices (PDIs), entrapment efficiencies (EEs), and drug loadings (DLs) are for optimized SLNs and NLCs.

**Table 3:** Effects of total lipid concentration on properties of SLNs and NLCs.

Lipid(s)	Lipid Concentration Changes	Results	Reference
Softisan^®^ 100	10–40 mg/mL	Increase in PS (~140–210 nm)	[36]
Tripalmitin, Phosal^®^ 53MCT	20–80 mg/mL	From 20–60 mg/mL: PS (~135–185 nm), PDI (~0.2, no change), EE (81.1–93.2%), and DL (9.20–10.43%) From 60–80 mg/mL: significant increases in PS (to ~235 nm) and PDI (to ~ 0.47), negligible changes in EE and DL	[76]
Glycerin monostearate	10–15 mg/mL	Increase in PS (320–360 nm)	[43]
Glycerol monostearate	50–100 mg/mL	Increase in PS (272–315 nm) and EE (69.7–80.9%)	[166]
Stearic acid	40–50 mg/mL	Increase in PS (282–305 nm) and PDI (0.32–0.69)	[169]

**Table 4:** Effects of liquid lipid level on the properties of NLCs.

Lipid(s)	Change of Liquid Lipid Level	Results	Reference
Tristearin, soya lecithin	20–50% *	Decrease in PS (426–311 nm) and PDI (0.38–0.24)	[160]
Tripalmitin, Phosal^®^ 53MCT	0–50% *	From 0–40%: decreases in PS (479–185 nm) and PDI (0.441–0.214), increases in EE (81.2–93.2%) and DL (9.21–10.43%) From 40–50%: significant increases in PS (to 213 nm) and PDI (to 0.521)	[76]
Glycerin monostearate, oleic acid	15–30 mg	Decrease in PS (210–194 nm) and PDI (0.355–0.242), increase in EE (83.9–93.3%)	[42]
Tristearin, distearoyl-phosphatidyl ethanolamine	33–67% *	From 33–50%: slight increase in PS (186–195 nm), decrease in PDI (0.388–0.265) From 50–67%: increase in PS (19–233 nm) and PDI (0.265–0.404)	[155]
Stearic acid, hosphatidylcholine	5–15 mg/mL	From 5–10 mg/mL: decreases in PS (283–234 nm) and PDI (0.57–0.41) From 10–15 mg/mL: increases in PS (234–247 nm) and PDI (0.41–0.68)	[169]

*: Liquid lipid amounts expressed as percentages of total lipid amounts.

**Table 5:** Effects of initial drug amounts on the properties of SLNs and NLCs.

Lipid(s)/Drug	Change of Initial Drug Amount	Results	Reference
Stearic acid, soya lecithin/Paclitaxel	0.05–0.25–0.5 mmol	PS (99–113–157 nm), EE (72.2–89.0–66.5%), DL (16.3–25.0–23.6%)	[165]
Stearic acid, soya lecithin, α-tocopherol/Paclitaxel	0.05–0.25–0.5 mmol	PS (90–96–129 nm), EE (58.6–75.4–53.0%), DL (12.0–31.5–18.1%)	[147]
Tripalmitin, Phosal^®^ 53MCT/Ondansetron hydrochloride	6.3–16.7% *	From 6.3–12.5%: PS (172–185 nm), PDI (0.191–0.214), EE (86.7–93.2%), and DL (5.14–10.43%) From 12.5–16.7%: PS (185–254 nm), PDI (0.214–0.509), EE (93.2–83.3%), DL (10.43–12.20%)	[76]
Tristearin, soya lecithin/Miconazole nitrate	2.5–10% *	From 2.5–7.5%: PS (322–328 nm), PDI (0.28–0.26), and EE (87.6–92.7%) From 7.5–10%: PS (328–353 nm), PDI (0.26–0.41), and EE (92.7–84.9%)	[160]
Tristearin, distearoyl-phosphatidyl ethanolamine/Paclitaxel	5–20% *	From 5–10%: PS (209–195 nm) and EE (74.1–84.5%) From 10–20%: PS (195–318 nm) and EE (84.5–75.5%)	[155]

*: Drug amounts expressed as percentages of total lipid amounts.
